# *Drosophila melanogaster p53* has developmental stage-specific and sex-specific effects on adult life span indicative of sexual antagonistic pleiotropy

**DOI:** 10.18632/aging.100099

**Published:** 2009-10-27

**Authors:** Morris Waskar, Gary N. Landis, Jie Shen, Christina Curtis, Kevin Tozer, Diana Abdueva, Dmitriy Skvortsov, Simon Tavaré, John Tower

**Affiliations:** ^1^ Molecular and Computational Biology Program, Department of Biological Sciences, University of Southern California, Los Angeles, CA 90089-2910, USA; ^2^ Department of Oncology, University of Cambridge Cancer Research UK Cambridge Research Institute, Li Ka Shing Centre, Robinson Way Cambridge CB2 0RE, England; ^3^ Current address: Department of Pathology and Laboratory Medicine, Children's Hospital Los Angeles, Keck School of Medicine, University of Southern California, Los Angeles, CA 90089-9034, USA; ^4^ Current address: Department of Human Genetics, UCLA School of Medicine, University of California, Los Angeles, USA; ^6^ Current address: Unilever Research Center, Bangalore 560066, India

**Keywords:** aging, sexual conflict, Geneswitch, maternal effects, tumor suppressor

## Abstract

Truncated and mutant forms ofp53 affect life span in Drosophila, nematodes and mice, however the role of wild-type p53 in aging remains unclear.  Here conditional over-expression of both wild-type and mutant p53 transgenes indicated that, in adult flies, p53 limits life span in females but favors life span in males. In contrast, during larval development, moderate over-expression of p53 produced both male and female adults with increased life span. Mutations of the endogenous p53 gene also had sex-specific effects on life span under control and stress conditions: null mutation of p53 increased life span in females, and had smaller, more variable effects in males. These developmental stage-specific and sex-specific effects of p53 on adult life span are consistent with a sexual antagonistic pleiotropy model.

## Introduction

**Figure 1.**
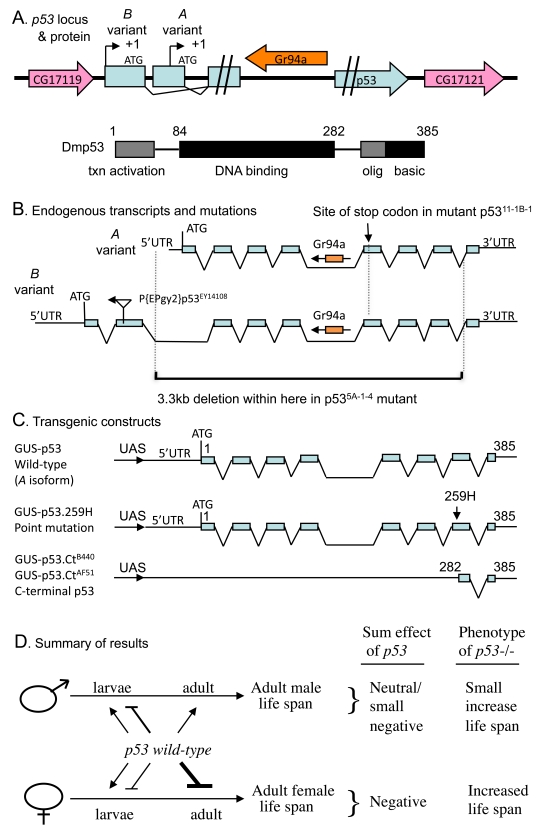
Summaryof *Drosophila p53* locus, mutations, transgenes and life span effects. (**A**) Diagram of *p53* locus and major protein product Dmp53.  The *p53* gene is indicated in blue, including the two promoters, indicated by black arrows.  The internal intron/exon structure of *p53* is omitted here for clarity, but is shown below in (**B**).  The pink arrows in indicate the genes that flank *p53* on the 5' and 3' side, genes *CG17119* and *CG17121*, respectively.  The orange arrow indicates the gustatory receptor gene *Gr94a*, located in the *p53* intron.  The 385 aa Dmp53 protein is diagrammed using black and gray boxes, including the N-terminal transcriptional activation domain, the central DNA binding domain, and the C-terminal oligomerization domain and basic region.  (**B**) Diagram of endogenous *p53* transcripts and mutations. The intron/exon structure of the A and B variant transcripts is indicated.  The Gr94a gene is indicated in orange with an arrow indicating orientation.  The location of insertion of the P element P{EPgy2}p53^EY14108^ in the second exon of the *B* isoform is indicated by a triangle, with an arrow indicating the orientation of the insert.  The lower black bracket indicates the breakpoints of the 3.3kb deletion in the p53[5A-1-4] mutation.  (**C**) Diagram of transgenic *p53* constructs.  (**D**) Summary of *p53* effects on adult life span.  The effect on adult life span of *p53* wild type (*A* variant) over-expression during larval development and in adults is diagrammed: Bars represent negative effects of *p53* wild-type on adult life span, while arrows represent positive effects on adult life span; thickness of the lines indicates relative strength of the effect. "Sum effect of *p53*" is the expected summation of effects of *p53* on adult life span, which is consistent with the life span phenotype of *p53* null mutation (*p53*-/-), as indicated.

The *p53* gene encodes a transcription factor that regulates apoptosis and metabolism and is mutated in the majority of human cancers [1,2]. The p53 protein functions as a tetramer with various protein domains mediating oligomerization, DNA  binding and  transcriptional transactivation.   *Drosophila* contains a single *p53* gene with a structure similar to humans [3-6] including two promoters, and the major protein products are of similar size: 393 amino acid residues for the human protein, Hp53, and 385 amino acid residues for the *Drosophila* protein, Dmp53 (*Drosophila* protein diagrammed in Figure 1A).  The  central DNA  binding  domain of Dmp53 protein shows partial sequence conservation with Hp53 [3]. The other domains of Dmp53 show less obvious sequence similarity to Hp53, but appear conserved in function. Similar to the N-terminal transcriptional activation domain of Hp53, the N-terminus of Dmp53 contains a high proportion of acidic residues, and Dmp53 has been shown to bind to conserved p53 response elements and activate transcription [3]. The C-terminus of Hp53 contains a basic region (9/26 residues) that can bind either DNA or RNA, and the C-terminus of Dmp53 is also relatively basic (6/24 residues).  Finally, the oligomerization domain is located in the C-terminal portion of Hp53, and the corresponding region of Dmp53 contains a conserved critical Gly "hinge" residue, and appears active in oligimerization based on yeast two hybrid assays.  The *p53* message is expressed at very low levels in adult tissues, with some enrichment indicated for the eye, malphigian tubule (similar to mammalian kidney), and female germ cells [7,8].

Mutant forms of p53 lacking function of a particular domain can have powerful dose-dependent effects that are often dependent upon the presence of wild-type p53 [3,9-11].  For example, specific truncated forms of mouse p53 can cause enhanced cancer resistance and accelerated aging phenotypes, generally interpreted as a state of p53 hyperactivation [12].  Based on studies in mammals it has been suggested that *p53* may exhibit antagonistic pleiotropy between life-cycle stages, in that it favors normal development, fecundity and cancer resistance in young animals, but may promote aging in old animals [9,13-15].  Recently *p53* gene activity was found to limit the life span of *C. elegans* hermaphrodites, and this effect was dependent upon the activity of the insulin/IGF1-like signaling (IIS) transcription factor gene *Daf-16/FOXO* [16].  In *Drosophila*, several dominant *p53* mutations and transgenes have been characterized, that generally appear to antagonize *p53* activity [3].  Nervous-tissue expression of one of these dominant *p53* transgenes (*p53* point mutation 259H) was found to inhibit IIS and extend life span in females [17,18].  However it remains unclear if and how *p53* might normally affect the life span of *Drosophila* males and females.  Here the wild-type form of *p53*, as well as mutant forms, were assayed for effects on *Drosophila* life span, in both male and female flies.

**Table 1.** 

*Strain #*	*Genotype *	*Group (notes)*
*2*	*w[1118] ; + ; Df(3R)Exel6193, P{XP-U}Exel6193 /TM6B, Tb (BL7672)*	*- (Chromosomal Def uncovers p53)*
*3*	*y[1] w[1118] ; + ; p53[5A-1-4] (BL6815) *	*- (deletion of p53 gene)*
*4*	*y[1] w[1118] ; + ; p53[11-1B-1] (BL6816) *	*M (pt mutant)*
*5*	*w[1118] ; p53[1] / TM6B, Tb*	*M (pt mutant)*
*6*	*w[1118] ; + ; +*	*M (the same pt mutant as line 4)*
*7*	*Oregon R ( + ; + ; +)*	*+*
8	*y[1] w[67c23]; P{EPgy2}p53[EY14108] (BL 20906) *	*+*
*9*	*w ; P{Switch}Actin 255B*	*(GeneSwitch Act-GS-255B driver)*
*16*	*y[1]w[1118]; P{w[+mC]=UAS-p53.Ex}3/T(2;3)TSTL, CyO:TM6B, Tb*	*(UAS-p53 wild type)*
*17*	*w ; P{w[+mC]=GUS-p53}2.1 *	*(UAS-p53 wild type - CDM26)*
*18*	*w; P{w[+mC]=GUS-p53.Ct}AF51*	*(C-terminal p53 - AF51)*
*19*	*w[1118]; +; P{w[+mC]=GUS-p53.Ct}B440/TM6B, Tb*	*(C-terminal p53 - B440)*
*20*	*w[1118]; P{w[+mC]=GUS-p53.259H}*	*(p53 point mutation - 259H)*

## Results

### Transgenic manipulation of *p53* in adult flies

**Figure 2.**
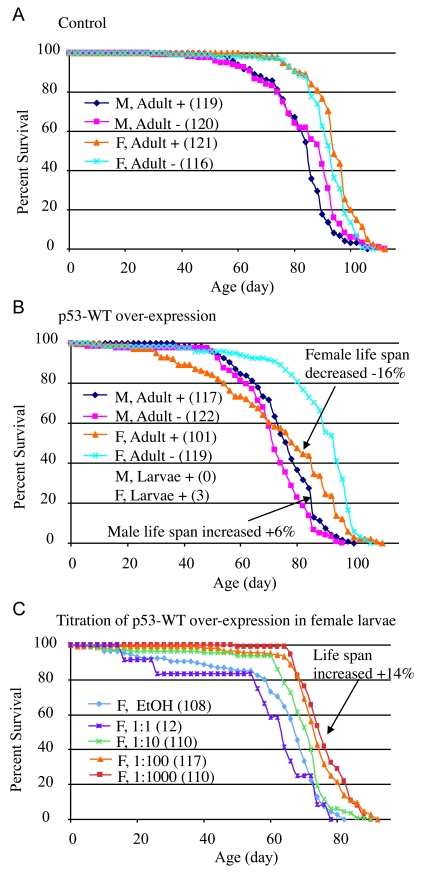
Conditional over-expression of wild-type *p53* trans-genes using Geneswitch system. All flies were the progeny of either Oregon R control (**A**) or p53-WT transgenic strain (**B**, **C**) crossed to the tissue-general Geneswitch driver Act-GS-255B.  The flies were cultured in the presence and absence of drug, as larvae or adults, as indicated: M =  males, F = females, + indicates culture in presence of drug, - indicates culture in absence of drug. The number of flies in each group are indicated in parentheses. (**A**, **B**) Blue diamonds indicate male adults plus drug, pink squares indicate male adults minus drug, orange triangles indicate female adult plus drug, turquoise x indicates female adults minus drug. (**A**) Control flies, progeny of Oregon R wild-type and Act-GS-255B.  (**B**) *p53* wild-type transgene over-expression.  Note male larvae plus drug produced no adult flies, whereas female larvae plus drug produced only three escapers.  (**C**) Titration of *p53* wild-type over-expression during female larval development and effect on subsequent adult life span. EtOH indicates the ethanol solvent for the drug alone (vector control, indi-cated with light blue diamonds).  Repeats of the titration experiments, including data for males are presented in Supplementary Figure 1.

*Drosophila p53* transgenes were assayed for effects on life span both in adults and during larval development (see below).  The conditional transgenic system Geneswitch  [19-21] was used to over-express both wild-type and mutant forms of *p53*.  With the Geneswitch system transgene expression is triggered by feeding flies (or larvae) the drug RU486/Mifepristone.  A Geneswitch driver strain called Act-GS-255B was used (Table 1, strain 9), where the tissue-general *actin5C* promoter drives expression of the Geneswitch transcription factor. In the presence of RU486, the  Act-  GS-255B driver produces expression of UAS-containing target constructs in all the tissues of either larvae or adults [19,22]: detailed characterization of the system using UAS-GFP reporter constructs demonstrates that the Act-GS-255B driver produces abundant transgene expression throughout all of the tissues of both adult flies and larvae, for both male and female animals, with slightly less (but still abundant) expression in adult males relative to females [22]. All of the flies examined in this study are the progeny of a cross; for example "16-9" flies are the progeny of a cross of males of strain 16 (containing the UAS-p53 wild-type transgene) with females of strain 9 (containing the Act-GS-255B Geneswitch driver) to generate progeny containing both constructs (strains summarized in Table 1); in all cases crosses are indicated with the male parent genotype first, and the female parent genotype second.  The RU486 drug itself had no significant effect on male or female life span when administered to adults (Figure 2A; statistical analyses summarized in Supplementary Table 1).  When wild-type *p53* was over-expressed specifically in adult flies, it had a negative effect (-16%) on mean life span in females (cross 16-9: 95% bootstrap CI for the ratio of the means [-21.11 - 11.61], log-rank p-value = 2.21 x10^-6^), and a positive effect (+6%) on mean life span in males (cross 16-9: 95% bootstrap CI [2.36 - 10.37], log-rank p-value  = 6.97 x10^-3^) (Figure 2B; Supplementary Table 1).  Slightly larger changes were observed for median life spans (Supplementary Table 1), and similar results were obtained with multiple independent transgenic insertions of *p53* wild-type (data not shown).  In contrast, adult-specific over-expression of the dominant mutant*p53* (point mutation p53-259H) transgene did not have a negative effect on female life span, and instead female life span tended to be increased (cross 20-9: +7%, 95% bootstrap CI [4.09 - 9.72], log-rank p-value = 4.05 x10^-8^) (Supplementary Figure 1B; Supplementary Table 1) [22], and similar results were obtained with *p53* dominant mutant transgene p53-Ct[B440] (Supplementary Figure 1C; Supplementary Table 1). Because these *Drosophila p53* dominant mutation transgenes are generally expected to antagonize the activity of wild-type *p53,* the data are consistent with wild-type *p53* having a negative effect on adult female life span. The negative effect on life span of wild-type *p53* over-expression in adult females and the lack of negative effect with dominant mutant *p53* transgenes was also confirmed using the FLP-*out* conditional system [23] to cause transgene over-expression (data not shown). Taken together, these data indicate that in adult flies, *p53* inhibits life span in females and favors life span in males.

## Transgenic manipulation of

A strikingly different set of results was obtained when *Drosophila p53* transgenes were expressed specifically during larval development.  When administered only during larval development, the drug RU486 itself had no effect on subsequent adult female life span, and a small negative effect on subsequent adult male life span (~-4%; Supplementary Table 1). Over-expression of wild-type *p53* at high levels during larval development was toxic to both males and females, in that no male adults were produced, and only three female adults (escapers) were obtained (Figure 2B). Intriguingly, the three female escapers had unusually long life spans: 86 days, 92 days, and 96 days, respectively. To determine if this apparent life span increase was significant, and to investigate the developmental effects of wild-type *p53* over-expression in greater detail, the over-expression was modulated by titration of the RU486/Mifepristone drug, in replicated experiments.  Titration of wild-type *p53* over-expression during development again indicated toxicity at high levels of expression, with greater toxicity evident for males (Supplementary Table 2).  Strikingly, at lower levels of induction, wild-type *p53* produced both female and male adults with increased mean and maximal life span (Figure 2C; Supplementary Figure 1E-F; Supplementary Table 2; female: +14%, 95% bootstrap CI [9.29 - 19.27]; log-rank p-value ≈ 0; male: +15%, 95% bootstrap CI [10.54 - 19.30]; log-rank p-value = 4.97 x 10^-7^).  These data demonstrate that high-level expression of *p53* can be toxic during development, whereas moderate over-expression of *p53* during development can cause increased life span in the resulting male and female adults.   Consistent with this conclusion, expression of the dominant mutant transgenes during development tended to decrease the life span of the resultant male and female adults (Supplementary Figure 1A-D, Supplementary Table 1).

### Effect of mutations in the endogenous *p53* gene

To confirm the effects of *p53* on *Drosophila* life span, flies were examined that had a deletion or mutation of the endogenous *p53* gene (mutations diagrammed in Figure 1B; strains listed in Table 1) [24]. Multiple trans-heterozygous *p53* wild-type and mutant allele combinations were assayed for life span simultaneously as a control for genetic background effects and environmental effects (the "L" cohort, data summarized in Supplementary Table 3, 4). This was done using two *p53* wild-type strains (called the "+" group; strains 6 and 7), two strains containing *p53* null mutation (called the "-" group; strains 2 and 3), and three strains containing  *p53* dominant mutations  (called the "M" group; strains 4, 5 and 8), and crossing each strain to each of the others in a "round-robin" approach.  In this way each of the various *p53* genotypes (+/+, -/-, +/-, +/M, -/M, M/M) represents the average of multiple specific genetic backgrounds.  This approach avoids the potential complication of identifying *p53* effects that might be specific to only one particular genetic background, such as would be created by using a backcrossing strategy.

**Figure 3.**
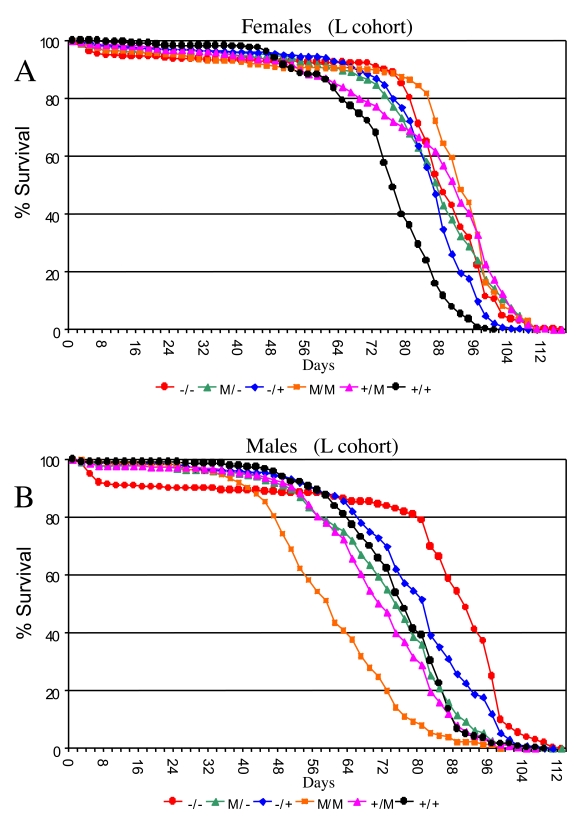
Effect of p53 mutations on life span. Cumulative survival curves for L cohort.   A key of p53 genotypes is presented below the graphs. Males are indicated with solid symbols and females are indicated with open symbols. (**A**) Females. (**B**) Males.

**Figure 4.**
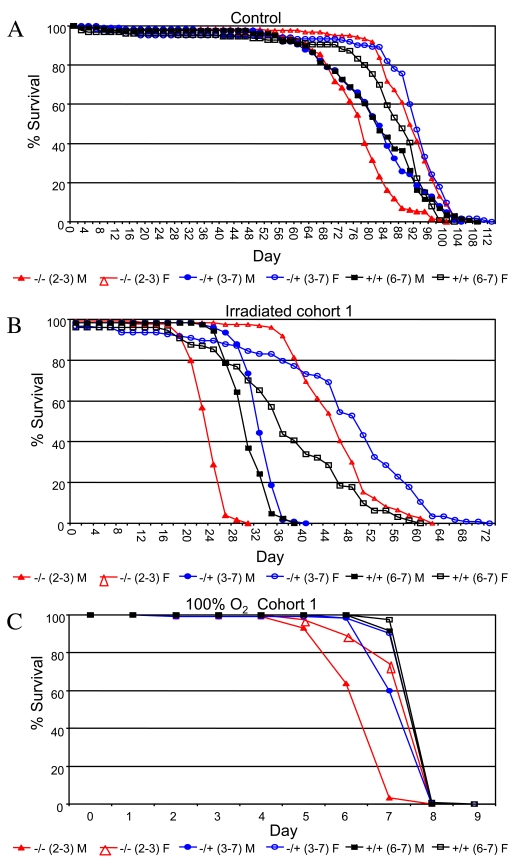
Survival curves for the indicated genotypes under stress conditions. (**A**) Ionizing radiation.  (**B**) 100% oxygen survival. A key of p53 genotypes is presented below the graphs. Males are indicated with solid symbols and females are indicated with open symbols. Survival curves for replicate experiments (cohort 2) are presented in Supplementary Figure 5. Survival statistics for these and replicate experiments are summarized in Supplementary Table 9.

In flies with mutations of the endogenous *p53* gene, the effect on life span should be the sum of the effects of *p53* at various life-cycle stages, both positive and negative (diagrammed in Figure 1D); and indeed, *p53* mutations were found to have a significant effect on life span in both sexes (ANOVA, p < 0.0001; Supplementary Table 5): Null mutation (-/-) of the *p53* gene increased mean female life span by +13% (95% bootstrap CI [9.00 -17.28]; log-rank p-value ≈ 0) relative to wild-type (+/+) controls (Figure 3A; Supplementary Figure 2A; Supplementary Table 4).  In the heterozygous *p53* mutant genotype (-/+) average female life span was also increased relative to wild-type controls by +11% (95% bootstrap CI [8.41 - 13.59]; log-rank p-value ≈ 0).  In male flies null mutation (-/-) of the *p53* gene increased mean life span by +12% (95% bootstrap CI [4.92-14.50]; log-rank p-value ≈ 0), whereas the effect of heterozygous mutation was smaller, yielding mean life span increases of +5.5% (95% bootstrap CI [2.15 - 7.53]; log-rank p-value ≈ 0) (Figure 3B; Supplementary Figure 2B; Supplementary Table 4). However, as seen below (Figure 4A, Supplementary Figure 4), the life span increases in *p53* mutant males were not consistently observed when crosses were done in the opposite direction, and therefore may not be biologically significant. Similar effects of *p53* null (-/-) and heterozygous (+/-) genotypes were obtained when the experiments were repeated using different culture conditions (richer food source and presence of mates) that yield shorter overall life spans (the "W" cohort; Supplementary Figure 3; Supplementary Table 6, Supplementary Table 7). Taken together, these data with endogenous *p53* gene mutations support the conclusion that, in sum, *p53* limits the life span of female flies, with smaller and more variable effects in male flies.

Several *Drosophila p53* dominant mutations (M) were examined and found to have complex effects on adult life span, depending upon the particular allele, and whether or not a wild-type copy of *p53* was present in the background (Figure 3; Supplementary Figure 2, Supplementary Figure 3).  Some of the variability in life span across genotypes is expected to result from differences in genetic background.  Indeed, the complexity of *p53* dominant mutations and their interactions with genetic background has recently been reviewed [25]. Strikingly, when the data for the various *p53* genotypes in the L cohort were grouped to control for genetic background effects, the dominant mutations tended to increase life span in females (+/M, -/M, M/M), and to decrease life span in males (+/M, M/M) (Figure 3; Supplementary Figure 2; Supplementary Table 4). Since the *Drosophila p53* dominant mutations are generally expected to antagonize wild type *p53* function, the increased life span of +/M females relative to wild type (+/+) is consistent with the results obtained above suggesting that, in sum, *p53* limits the life span of females. However, for the M/M genotype flies, a wild-type copy of the entire *p53* gene is not present, and these genotypes produced the greatest increase in life span in females and the greatest decrease in life span in males.  Therefore, these data suggest that the mutant forms of *p53* may have sexually antagonistic effects on *Drosophila* life span that are not necessarily dependent upon the presence of a wild-type *p53*. Strikingly, these effects of dominant mutations on life span were highly dependent upon environment, since in the W cohort the dominant mutations tended to decrease life span in both males and females (Supplementary Figure 3; Supplementary Table 7). It will be of interest in the future to determine what is the mechanism for these opposite effects of dominant *p53* mutations in males versus fe-males, and to determine if the dramatic gene-by-environ-ment effect of *p53* dominant mutations in females is due to the presence of mates, the richer food source, or both.

### Controls for maternal effects and *X* chromosome effects

In an effort to control for possible maternal effects and *X* chromosome effects, several life span assays were repeated with the crosses done in both directions simultaneously, i.e., varying which strain serves as mother or father for the cross (Supplementary Figure 4).  An increase in life span of *p53* null mutant (-/-) flies relative to wild-type (+/+) controls was obtained in female progeny regardless of cross direction (Supplementary Figure 4; Supplementary Table 8), thereby ruling out a primary effect of maternal genotype. In males a consistent change in life span was not observed, in that although the null mutants exhibited slight differences in life span compared to controls, the direction of change differed depending on the direction of the cross. Furthermore, while the survival curves of many of the reverse cross pairs differed from one another in both sexes (log-rank test, data not shown), in females there was strong concordance and highly significant results from comparisons of survival curves in both cross directions and relative to both controls, while this was not the case for males (Supplementary Table 8).  These results demonstrate that the increased life span in females due to *p53* mutation cannot be simply due to maternal or *X* chromosome effects, and in conjunction with the above findings, these data again suggest that *p53* preferentially limits the life span of female flies.

### Sex-specific effects *p53* on fly stress resistance

*Drosophila p53* is required for normal resistance of larval cells and tissues to certain kinds of stress, for example, ionizing radiation and UV toxicity [26,27], and third-instar larvae that are null for *p53* exhibit decreased survival when challenged with 4,000 Rads of ionizing radiation [28].  To determine if *p53* genotype might have sex-specific effects on stress resistance in adult flies, male and female flies that were either wild-type or mutant for *p53* were subjected to two types of life-shortening stress, ionizing radiation and 100% oxygen atmosphere, in replicated experiments (Figure 4, Supplementary Table 9).  Treatment with 90,000 Rads of gamma-irradiation on day 10 of adult age reduced adult life spans by half, and *p53* mutant female flies were again found to have greater mean life span than wild-type controls (+/-: +18%, 95% bootstrap CI [13.13 - 23.36]; log-rank p-value = 0;  -/-: +13%, 95% bootstrap CI [9.09 - 16.71]; log-rank p-value = 2.98 x10^-4^).  In contrast, *p53* mutations were found to slightly reduce the survival of female flies subject to 100% oxygen atmosphere (-/+: not significantly different than wild-type; -/-: -4%, 95% bootstrap CI [-5.06 - -3.34]; log-rank p-value = 1.28 x10^-13^). In males, *p53* null mutants subject to ionizing radiation had significantly reduced mean life span, whereas heterozygotes fared slightly better than wild-type (+/-:  +4%, 95% bootstrap CI [1.80 - 6.00]; log-rank p-value = 2.02 x10^-7^; -/-: -19%, 95% bootstrap CI [-20.68 - -17.06]; log-rank p-value  ≈ 0). As with females, *p53* gene mutations tended to reduce male survival in response to a 100% oxygen environment (+/-: -4%, 95% bootstrap CI [-4.38 - -3.05]; log-rank p-value  = 4.44 x10^-16; ^-/-: -15%, 95% bootstrap CI [-16.13 - -14.10]; log-rank p-value  ≈ 0). Therefore, wild-type *p53* tended to favor the survival of both sexes under 100% oxygen stress conditions, yet was detrimental to female life span in flies subject to ionizing radiation. Therefore the results for adults subject to ionizing radiation were similar to those observed during normal aging: normal *p53* function increased survival of males and decreased survival of females. The fact that *p53* favored the survival of both sexes under the more severe life-shortening condition of 100% oxygen stress may be indicative of a threshold effect on survival that is sex-specific.

## Discussion

In these experiments a combination of genetic and transgenic approaches were used to study how *p53* affects the life span of male and female *Drosophila*.  The conditional transgenic system Geneswitch was employed to produce tissue-general expression of *p53*, either during development or specifically in adults.  Detailed characterization of the Geneswitch driver strain ("Actin-GS-255B") using GFP reporter constructs demonstrated that the system yields truly tissue-general expression during larval development, as well as tissue-general expression in both male and female adults [22].  The data indicate that *Drosophila p53* has effects on adult life span that are antagonistically pleiotropic between developmental stages and sexes (summarized in Figure 1A).  One advance of the present study is that life span effects were identified using transgenes encoding the full length, wild-type form of *Drosophila* p53 protein, as well as ones encoding mutant forms. In adults, wild-type *p53* over-expression limited life span in females and favored life span in males. In contrast, during development, *p53* over-expression acted in a dose-dependent manner to either reduce or increase the subsequent longevity of both male and female adults: high level expression during development was detrimental, whereas moderate over-expression produced increased life span. The dominant mutation transgenes generally produced the opposite effect of wild type *p53 *transgenes, in both males and females.  This indicates that the opposing effects of *p53* transgenes on male and female life span cannot be simply due to some cryptic difference in the efficiency of transgene expression in males versus females, or to some differential toxicity of the encoded proteins in males versus females.

Results consistent with the transgenic manipulations were obtained from analysis of the endogenous *p53* gene:  Null mutation of the endogenous *p53* gene increased life span in females, and had smaller, more variable effects on male life span.  The effects of *p53* on adult fly survival under stress conditions were also sex-biased:  wild-type *p53* was found to favor the survival of both sexes under 100% oxygen stress conditions, yet to be detrimental to female life span in flies subject to ionizing radiation. In these experiments *p53* expression and function is being altered in all of the tissues of the animal simultaneously, and therefore the effects observed are the sum of any possible tissue-specific effects of *p53*. Indeed our results suggest that the positive and negative effects of *p53* on life span observed here with tissue-general alterations are comprised of a mix of both positive and negative tissue-specific effects, that combine to result in the observed opposite effects in males versus females (J.S. and J.T., 2009 Experimental Gerontology, in press).

The data presented here indicate that *p53* null mutation increases life span in female flies, with smaller, more variable increases observed for male flies.  Helfand and coworkers have previously reported that *p53* null mutant male and female flies were sickly, with a shortened life span, however, statistical analysis was not presented [17]. One possibility is that the apparent reduction in life span and vigor previously reported for *p53* null flies may have resulted from inbreeding depression in the homozygous mutant flies used in that study.  In contrast, in the experiments presented here, multiple trans-heterozygous *p53* null mutant genotypes were examined, so as to reduce possible inbreeding effects, and thereby reveal the life span benefit of *p53* null mutations.  Helfand and coworkers also analyzed the effect on life span of nervous system-specific expression of two *p53* dominant mutant transgenes, a C-terminal fragment transgene (p53-Ct), and the point mutant (p53-259H).  They found that nervous system expression of p53-Ct throughout both development and adulthood increased female life span by +58%, and increased male life span by +32% [17].  Because the dominant mutations are generally expected to antagonize p53 activity, their results are consistent with our conclusion that, in sum, *p53* limits life span in females, with smaller effect in males (summarized in Figure 1D). Using the Elav-Geneswitch driver to restrict expression to the adult nervous system, Helfand and coworkers found that the p53-Ct transgene increased female life span by +18% to +26%, and the p53-259H transgene increased female life span by +11% to +13%, again consistent with our finding that *p53* limits the life span of adult females.  Indeed, using the tissue-general Act-GS-255B driver to restrict transgene expression to adults, we also found that the p53-Ct and p53-259H transgenes produced an increase in median life span in females (Supplementary Figure 1A-D) [22].  For adult-specific expression in male nervous system, Helfand and coworkers reported life span data for only two assays, both using the p53-Ct transgene: using a high-calorie food condition, male life span was reported to be increased by +13%, whereas using a low-calorie food, male life span was unchanged, and results for normal food were not presented [17].  That result might at first appear to be partly inconsistent with our conclusion that *p53* favors life span in adult males, however, there are several possible explanations that might reconcile these results. First, the previous experiment involved the p53-Ct transgene, encoding the p53 C-terminal fragment, and data from mammals suggests that certain dominant p53 mutants are capable of either antagonizing or promoting p53 activity, depending upon the level of expression and the cellular context [11].  Second, the life span increase was observed only under a high-calorie food condition, and our data suggest sex-specific interactions between dominant *p53* mutations and diet/environment with regard to life span (Figure 3, Supplementary Figure 2). Under our conditions and using tissue-general expression, we found that adult-specific expression of the dominant mutant *p53* transgenes tended to decrease male life span (Supplementary Figure 1, Supplementary Table 1), consistent with our conclusion that *p53* normally favors adult male life span. Finally, the effects of tissue-general expression, as tested here, will be the sum of all tissue-specific effects, be they positive or negative. Indeed our results suggest that the positive and negative effects of *p53* on life span observed here with tissue-general alterations are comprised of a mix of both positive and negative tissue-specific effects (J.S. and J.T., 2009 Experimental Gerontology, in press), that combine to result in opposite effects in males versus females (summarized in Figure 1D). Therefore, the previous results from the Helfand group (with the possible exception of a single assay of males under a high-calorie food condition), are generally consistent with the results presented here.

One possible mechanism by which *p53* might act in adult flies to preferentially limit female life span is by stimulating IIS, since IIS appears to preferentially limit life span in females of *Drosophila* and other species [29,30]. Studies in mammals provide precedent for crosstalk between *p53* and the IIS pathway, including the target transcription factor FOXO, in regulating both aging and cancer [31,32].  Consistent with this idea, life span extension in *Drosophila* females produced by nervous system-specific expression of the dominant mutant *p53-259H* transgene was found to correlate with a reduction in IIS signaling [18]. In *C. elegans*, mutation of the *p53* homolog *cep-1* increased life span of adult hermaphrodites, and this increase required the function of the IIS target transcription factor gene *Daf-16/FOXO* [16]. To definitively rule in (or out) a role for IIS in *Drosophila p53* life span effects will require future assays in the presence and absence of the Foxo transcription factor.

Another possible mechanism by which *p53* might affect life span is by altering proliferation or causing apoptosis in particular cell types.  For example, ablation of germ-line cells in adult animals by forced over-expression of the *bam* gene caused increased life span in males and females [33]. However, while germ line ablation might be attractive as a possible mechanism for the increased life span observed in *p53*-over-expressing males, it is not consistent with the life span decrease observed in females. Alternatively, over-expression of wild-type *p53* specifically in adult diploid cells using an *escargot*-GAL4 driver caused ablation of most stem cells in the gut, and gut stem cell proliferation appears to be more rapid in females than in males [34].  While this might be attractive as a possible mechanism for the life span decrease observed in *p53*-over-expressing females, it is not consistent with the life span increase observed in males; indeed other experiments involving disruption of adult diploid cell function caused an equally dramatic decrease in life span in both sexes [35].  It will be of interest in the future to ask if *p53* might be affecting life span through highly sex-specific or sexually opposite effects on cell proliferation and survival.  Notably, over-expression of strong caspase inhibitors and other apoptosis and senescence regulatory genes in adult flies did not yield increased life span in either sex, and where negative effects on life span were observed, such as with *wingless* and *activated Ras*, the negative effects were similar in males and females [22].  Those results tend to suggest that *p53* may be acting through some other mechanisms, such as alterations in metabolism or autophagy.  Additional possible mechanisms by which *p53* might affect life span include sex-specific alterations in behavior, such as food intake, or potentially costly activities such as movement or aggression.

In these experiments* Drosophila p53* was also found to have sex-specific effects on survival under stress conditions.  Wild-type *p53* favored the survival of both sexes under 100% oxygen stress, yet was detrimental to female life span in flies subject to ionizing radiation.  This may be indicative of a threshold effect on survival that is sex-specific. Mechanistically the ability of *p53* to either favor survival or mortality may be related to *p53*'s ability to regulate both repair and apoptotic pathways [1,36-38], and perhaps the functional connection between *p53* and FOXO in response to oxidative stress [25]. In line with our findings, *C. elegans* hermaphrodites that are long-lived due to *p53* (*cep-1*) mutation did not demonstrate increased resistance to oxidative (or UV) stress [16], however resistance to gamma irradiation was not examined. Strikingly, in *C. elegans* hermaphrodites, *p53* has recently been found to increase life span in response to mild mitochondrial stress, and to decrease life span in response to severe mitochondrial stress, consistent with a threshold effect on survival [39] ; however effects in males have not been reported.  In mice, reduced *p53* function results in resistance to lethality caused by moderate gamma irradiation and increased sensitivity to severe irradiation [40,41], again suggestive of a threshold effect, however any potential sex-bias has not been reported.  Finally, long-lived female *Drosophila* that over-expressed dominant-mutant *p53* in neurons exhibited increased resistance to the oxidative stressor paraquat [17]; however effects in males were not reported. Taken together the data are consistent with a model in which*p53* has a threshold effect on survival under stress, and the threshold for the transition from favorable to detrimental depends upon the type of stress and the sex of the animal.  Such a threshold model is consistent with extensive data from mammals and model systems demonstrating that *p53* can either favor oxidative stress resistance and cell survival, or favor oxidative stress and cell death, depending upon the cellular and environmental context, and the degree of activation of *p53* [38]. In mammals, physiological levels of *p53* activity appear to maintain normal cellular redox status, through sustained expression of antioxidant genes (e.g., *Sesn1&2, GPX1, AIF*) and metabolic genes (e.g., *SCO2, PGM, TIGAR*).  In contrast, hypo-physiological levels of *p53* activity can suppress expression of antioxidant genes (e.g., *Sesn1&2, GPX1*) and cause increased oxidative stress. Similarly, hyper-physiological levels of *p53* activity can induce pro-oxidant and apoptosis-promoting genes (e.g., *NQO1, POX, BAX, PUMA, p66shc*), and/or cause an imbalance in expression of antioxidant genes (e.g., *MnSOD, PIG12, ALDH4, GPX*), and again cause increased oxidative stress [38].

Antagonistic pleiotropy of gene function between younger and older animals is generally accepted as one of the most likely genetic mechanisms underlying aging [42]; however, specific genes exhibiting such pleiotropy have generally not been identified.  One notable exception is data from mammals that suggests *p53* exhibits antagonistic pleiotropy between developmental stages.  At young ages *p53* favors fecundity and favors survival by acting as a tumor suppressor, yet at late ages it may limit survival by promoting cell senescence, or through other mechanisms [13,43]. Increasing evidence suggests that genes can also exhibit antagonistic plieotropy of function between the sexes, affecting a variety of traits including reproductive fitness and life span [30,44-47]. The data presented here suggest that *Drosophila p53* exhibits a combination of both developmental stage-specific and sex-specific antagonistic pleiotropy with regard to life span. If this result were to translate to humans, it would have implications for human aging related diseases such as cancer. Consistent with our results using flies, the effects of human *p53* and *p53*-interacting genes such as *MDM2* on cancer incidence and longevity are often sex-biased [48], and *p53* has recently been implicated in regulating mammalian maternal fecundity [49]. Moreover, during mouse development, *p53* null mutations cause a high frequency of neural tube defects and lethality that preferentially affects female embryos [50,51], and interestingly, this sex difference appears to result from the number of *X* chromosomes rather than the presence or absence of the *Y* [52].  The sex-specific effects of*p53* may be related to recent observations that in humans the *X*-chromosome dosage-compensation gene *MOF* can regulate *p53* [53]; and notably the *MOF* gene is conserved and also *X*-linked in flies. Taken together the data support a sexual antagonistic pleiotropy model in which *p53* function may be maintained by positive selection for fecundity and/or survival benefit during development, in young animals, and under certain stress conditions, despite acting at another stage of the life cycle and in the other sex to limit adult life span (summarized in Figure 1D).

## Methods

*Drosophila* culture. Drosophila culture and life span assays were performed as previously described [19]. Briefly, crosses were conducted in 250 ml urine-specimen bottles (Genessee Scientific) containing 35 ml of medium. Adult flies were maintained in narrow polystyrene vials (Genesee Scientific) containing 5 ml medium. Drosophila culture media contained cornmeal, agar, dextrose, yeast, and propionic acid to inhibit bacterial growth and tegosept to inhibit fungal growth [54]; except for the W cohort which were cultured on an older recipe containing molasses rather than dextrose (food recipes summarized in Supplementary Table 10). Flies were maintained at 25^o^C and on a 12:12 dark/light cycle, and were removed to room temperature for less than 1 hour every 2 days to provide fresh medium and remove and enumerate dead flies. To estimate life expectancy, single-sex mortality vials were established, with ~25 flies per vial (sample sizes were occasionally reduced due to rare escapers) and 5 or 10 replicate vials (depending on the experiment) per sex for every cohort.  The L cohort deletion experiment used 10 replicate vials per sex, the reverse-cross experiments used 5 vials per sex, the stress experiments used 5 vials per sex, the Geneswitch experiments used 5 vials per sex, and the drug-titration experiments used 5 vials per sex. Note that for each line in the W cohort ~125 flies were maintained at ~25 flies per vial with mates.

*Drosophila* strains . All *Drosophila* strains and genotypes are listed in Table 1, and several mutants and transgenes are diagrammed in Figure 1. Wild-type (*A*-isoform) and dominant-mutant *p53* transgene stocks were obtained from Michael Brodsky [3] and Bloomington *Drosophila* Stock Center.  P{UAS-p53.Ex}, *p53* wild-type. P{GUS-p53.Ct}AF51, C-terminal fragment AA285-385, chromosome 2. P{GUS-p53.Ct}B440, C-terminal fragment AA285-385, chromosome 3. P{GUS-p53.259H}, AA substitution, chromosome 3.  The *p53* mutant strains were obtained from Kent Golic and Bloomington *Drosophila* Stock Center [55].  Df(3R)slo3 is deletion of entire *p53* gene ("-"). Df(3R)Exel, P{XP-U}Exel is deletion of entire *p53* gene ("-"). *p53*[5A-1-4] is 3.3kb internal deletion ("-"), and it's structure was confirmed by PCR amplification and sequencing (diagrammed in Figure 1B).  *p53*[11-1B-1] is a point mutation that introduces a stop codon at nucleotide residue 211, and is predicted to yield a 70AA truncated protein ("M").  P{EPgy2}p53[EY14108] is a P element insert mutation obtained from Bloomington *Drosophila* Stock Center (BL 20906), and the insertion was mapped to the first exon of the *p53* B-variant using inverse PCR (diagrammed in Figure 1B) [56].  Because the p53[EY14108] mutation is predicted to produce an altered complement of p53 protein isoforms, it is grouped here with the dominant mutants ("M").

Geneswitch conditional gene expression system .  Geneswitch strains and protocols are as previously described [19-21].  The strain Act-GS-255B [19,22] contains two inserts on the second chromosome of a construct in which the actin5C promoter drives expression of the Geneswitch coding region.  RU486 (Mifepristone, Sigma) was fed to adult flies or developing larvae by adjusting the food to ~160ug/ml final concentration.  A stock solution of 3.2mg/ml of RU486 was prepared by dissolving drug in ethanol (100%). Control food received ethanol solvent alone.  In certain experiments RU486 concentrations were titrated as indicated.  All ages are expressed as days from eclosion at 25^o^C. To generate flies containing both the Act-GS-255B driver and the UAS-transgenes, virgins from the Act-GS-255B strain were crossed to males from each transgenic strain and the Oregon R wild-type strain as a control.  Certain crosses were done in the opposite direction, as indicated in the "reverse cross" experiments.  The life span assay result for p53-259H transgene over-expression in adult flies using Act-GS-255B driver has been previously published [22], and is included here with additional statistical analysis for comparison purposes (Supplementary Table 1).

Statistical analyses .  Initial cohort size was taken to be the number of flies in the vials at the beginning of the second two-day interval. Deaths during the first interval after transfer were considered to be due to injury during collection and therefore were excluded from the calculations. Survivorship was scored every other day and final cohort size was taken as summed deaths. The effect of*p53* deletion, mutation, and over-expression on *Drosophila* life span was assayed in multiple trials for several lines. Life span summary statistics for each of the experiments (data pooled across replicate vials) and detailed statistical analyses are presented in the Supplementary Materials (Supplementary Table 1-9).  A non-parametric log-rank test was employed to compare the survival functions between*p53* deficient or over-expression genotypes and controls [57]. To further assess the effect of *p53* on mean, median, and "maximal lifespan" (defined operationally here as the 90^th^ percentile of life span), 95% double bootstrap-t confidence intervals for the ratio of the means (or ratio of the percentiles) of the experimental and control samples were computed using a custom Fortran script. Mixed effects models were fit to data from each sex separately to ascertain the effects of mutation type (M) and genotype (G) (fixed main effects) on life expectancy, with replicate vials (R) treated as a random effect using the *nlme* package in R.  Mixed-effects models allow for a flexible representation of the covariance structure due to the grouping of the data and enabled the variation induced in the survival response by replicate vials to be characterized. As appropriate, the models were y = μ + M + R(M) + ε (where M = +/+, +/-, etc and G = 6-7, 2-6, etc was treated as an "inner" grouping) and y = μ + G + R(G) + ε, where ε indicates the within vial error variance. Post-hoc Tukey tests were performed to assess significant differences among means after correcting for multiple testing. Analyses were performed using the R statistical environment [58], unless otherwise noted.

## Supplementary figures

Supplementary Figure 1 Conditional over-expression of wild-type and dominant-mutant p53 transgenes using Geneswitch system. All flies were the progeny of the indicated transgenic strains crossed to the ubiquitous Geneswitch driver Act-GS-255B. The flies were cultured in the presence and absence of drug, as larvae or adults, as indicated: M = males, F = females, A = adults, L = larvae, "+" indicates culture in presence of drug, "-" indicates culture in absence of drug.  (**A**) Controls: progeny of Act-GS-255B driver crossed to Or-R wild type.  (**B-D**) p53 dominant-mutant transgene over-expression. (B) UAS-p53-259H.  (**C**) UAS-p53-B440.  (**D**) UAS-p53-AF51. (**E-G**). Titration of p53 wild-type (UAS-p53WT-CDM26) over-expression during development and effect on subsequent adult life span. (**E**) Males, cohort 1. Females of cohort 1 are shown in Figure 2.  (**F**) Females, cohort 2.  (**G**) Males, cohort 2.

Supplementary Figure 2 Survival data for each genotype in cohort L. Survival curves.(**A**) Females. (**B**) Males.

Supplementary Figure 2C Survival data for each genotype in cohort L. Survival curves. (**C**) Box plot presentation of survival data for each genotype in cohort L. Blue boxes indicate males, pink boxes indicate females.

Supplementary Figure 3 Survival curves for flies in cohort W. Grouped data. (**A**) Females.  (**B**) Males.

Supplementary Figure 3C Survival curves for flies in cohort W. Survival curves for each genotype.  (**C**) Females. (**D**) Males

Supplementary Figure 4 Reciprocal crosses. (**A**) Females.  (**B**) Males.

Supplementary Figure 4C Reciprocal crosses. (**C-G**) Comparisons of reciprocal crosses for specific genotypes. X and Y chromosomal composition of the flies is summarized to the right, along with the maternal p53 genotypes.

Supplementary Figure 4H Reciprocal crosses. (**H**) Box plot presentation of survival data for reciprocal crosses. Blue boxes indicate males, pink boxes indicate females.

Supplementary Figure 5 Survival data for flies subjected to stress. (**A**) Irradiation, cohort 2.  (**B**) 100% oxygen atmosphere, cohort 2.

Supplementary Figure 5C Survival data for flies subjected to stress. Box plot presentation of survival data for flies subjected to stress; data is the sum of cohorts 1 and 2.  (**C**) Irradiation.  (**D**) 100% oxygen atmosphere. Blue boxes indicate males, pink boxes indicate females.

## Supplementary tables

Supplementary Table 1 Summary of the effect on life span of wild-type p53 over-expression using the GeneSwitch system. 95% double bootstrap-t confidence intervals for the ratio of the means (or ratio of the percentiles) of the mutant and wild-type samples were computed as listed. The mean, median, and maximal life span values are reported for each genotype as well as the P-values representing the significance of the log-rank test of the null hypothesis that there is no difference in the probability of death between functions between wild-type untreated and p53 over-expressing flies. Note that * indicates 1.00 x10^-3^ <  P < 5.00 x10^-2^, ** indicates  1.00 x10^-8^ P < 1.00 x10^-3^, *** indicates P < 1.00 x10^-8^.

Supplementary Table 2 Summary of the effect of wild-type p53 over-expression titrated at various levels during development on Drosophila life span. Wild-type p53 over-expression was induced using the GeneSwitch system and titrated at various levels with the drug RU486. Note that for the 1:1 dilution, only 1 male pupae enclosed. 95% double bootstrap-t confidence intervals for the ratio of the means (or ratio of the percentiles) of the mutant and wild-type samples in each condition were computed as listed for each p53 concentration in the combined data from two trials. The mean, median, and maximal lifespan values are reported for each genotype as well as the P-values representing the significance of the log-rank test of the null hypothesis that there is no difference in the probability of death between functions between wild-type untreated and p53 over-expressing flies. Note that * indicates 1.00 x10^-3^ <  P < 5.00 x10^-2^, ** indicates  1.00 x10^-8^ P < 1.00 x10^-3^, *** indicates P < 1.00 x10^-8^.

Supplementary Table 3 Summary of the significance of p53 deletion or mutation on life span. To assess the effect of p53 mutation on mean, median, and maximal lifespan, 95% double bootstrap t confidence intervals for the ratio of the means (or ratio of the percentiles) of the mutant and wild-type samples were computed as listed for the combined data for the L-cohort and stress assays. The log-rank test was employed to test the null hypothesis that there is no difference in the probability of death between wild-type and p53 mutant flies. P-values indicating the significance of the tests are reported. ⊗Indicates exclusion of an outlier vial.

Supplementary Table 4 Summary of the effect p53 deletion or mutation on life span in grouped data. To assess the effect of p53 mutation on mean, median, and maximal lifespan, 95% double bootstrap t confidence intervals for the ratio of the means (or ratio of the percentiles) of the mutant and wild-type samples were computed as listed for the grouped L-cohort data. The mean, median, and maximal lifespan values are reported for each genotype as well as the P-values for the log-rank test of the null hypothesis of identical survival functions between wild-type and p53 mutant flies.  Note that * indicates 1.00 x10^-3^ <  P < 5.00 x10-2, ** indicates  1.00 x10^-8^ P < 1.00 x10^-3^, *** indicates  P < 1.00 x10^-8^. ⊗Indicates exclusion of an outlier vial.

Supplementary Table 5 Effect of p53 mutation on Drosophila life span. ANOVA results for differences in mean life span in Drosophila with differing p53 mutation types, where the main effect is the mutation type, comprised of grouped genotypes, and replicate vials are treated as a random effect in males (a) and females (c). Similar tests were also performed where the main effect is genotype in males (b) and females (d). Significant differences in group means were identified using Tukey's Honestly Significant Difference (HSD) multiple comparison and adjusted p-values based on the single-step method are reported for the relevant comparisons of various mutation types to wild-type.

Supplementary Table 6 Summary of the significance of p53 deletion or mutation effects on life span in W cohort. To assess the effect of *p53* mutation on mean, median, and maximal lifespan, 95% double bootstrap t confidence intervals for the ratio of the means (or ratio of the percentiles) of the mutant and wild-type samples were computed as listed for the W-cohort. The log-rank test was employed to test the null hypothesis that there is no difference in the probability of death between wild-type and *p53* mutant flies. P-values indicating the significance of the tests are reported.

Supplementary Table 7 Grouped life span data from W cohort experiments with log rank, average, standard deviations, medians and standard deviations of medians. ^a^   Mean life span, days +/- SD. ^b^  Median life span, days +/- SD Life Span, days.

Supplementary Table 8 Summary of the effect of p53 deletion or mutation on life span for the reverse-cross data. The mean, median, and maximal lifespan values are reported for each genotype as well as P-values for the log-rank test of the null hypothesis of identical survival functions between wild-type (+/+; 6-7) or the reverse cross wild-type (+/+; 7-6) and *p53* mutant flies are denoted by superscript a and b, respectively. ⊗ Indicates exclusion of an outlier vial.

Supplementary Table 9 Summary of the effect of p53 deletion on life span when flies were subject to ionizing radiation or a 100% oxygen environment. 95% double bootstrap-t confidence intervals for the ratio of the means (or ratio of the percentiles) of the mutant and wild-type samples in each condition were computed as listed. The mean, median, and maximal lifespan values are reported for each genotype as well as the P-values representing the significance of the log-rank test of the null hypothesis that there is no difference in the probability of death between wild-type and *p53* mutant flies.

Supplementary Table 10 Summary of food (fly culture media) recipes. The W cohort was cultured on "Old food" recipe, as were all flies in experiments in Tower laboratory prior to September 2005.  The L cohort and all other experiments presented here were conducted using "New food" recipe.
