# Supplementary figures and images for: Drosophila melanogaster p53 has developmental stage-specific and sex-specific effects on adult life span indicative of sexual antagonistic pleiotropy
Source: Aging (Albany NY). 2009 Oct 27;1(11):903–36. doi: 10.18632/aging.100099 (PMC2815744; doi:10.18632/aging.100099)

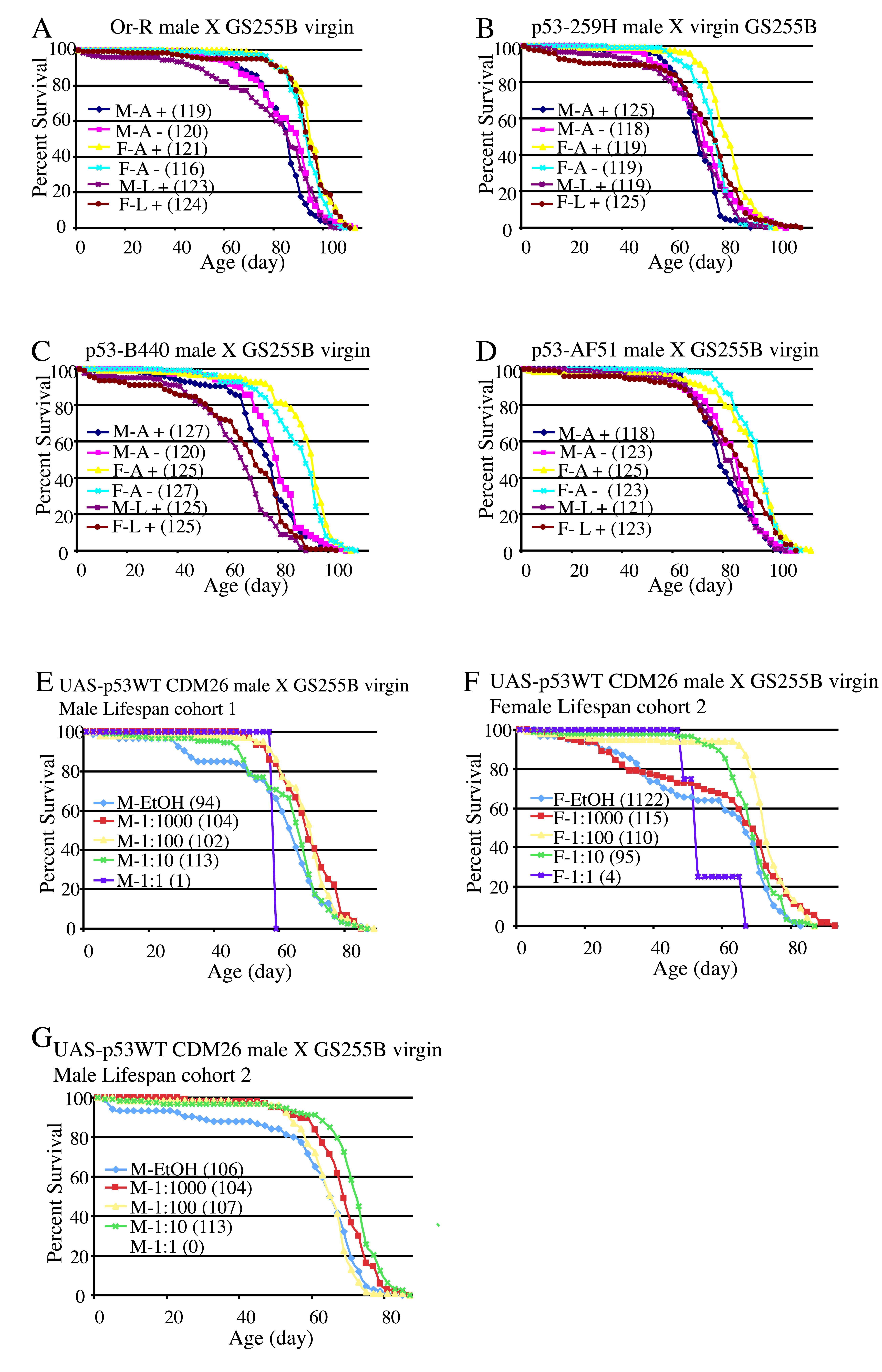

Supplement: Supplementary Figure 1 — All flies were the progeny of the indicated transgenic strains crossed to the ubiquitous Geneswitch driver Act-GS-255B. The flies were cultured in the presence and absence of drug, as larvae or adults, as indicated: M = males, F = females, A = adults, L = larvae, "+" indicates culture in presence of drug, "-" indicates culture in absence of drug. (A) Controls: progeny of Act-GS-255B driver crossed to Or-R wild type. (B-D) p53 dominant-mutant transgene over-expression. (B) UAS-p53-259H. (C) UAS-p53-B440. (D) UAS-p53-AF51. (E-G). Titration of p53 wild-type (UAS-p53WT-CDM26) over-expression during development and effect on subsequent adult life span. (E) Males, cohort 1. Females of cohort 1 are shown in Figure 2. (F) Females, cohort 2. (G) Males, cohort 2. [file aging-01-903-s001.tif]

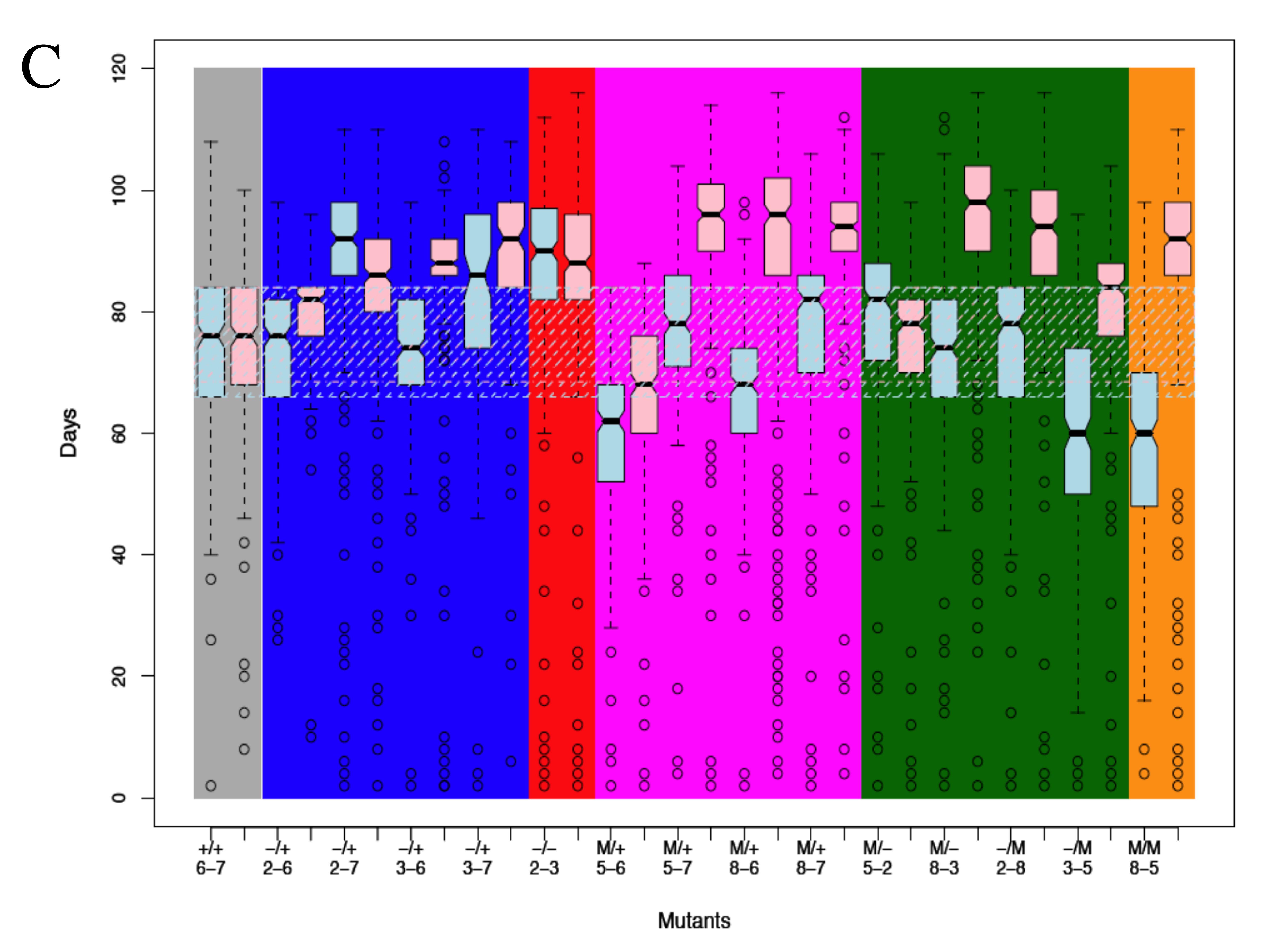

Supplement: Supplementary Figure 2 — Survival curves.(A) Females. (B) Males. [file aging-01-903-s002.tif]

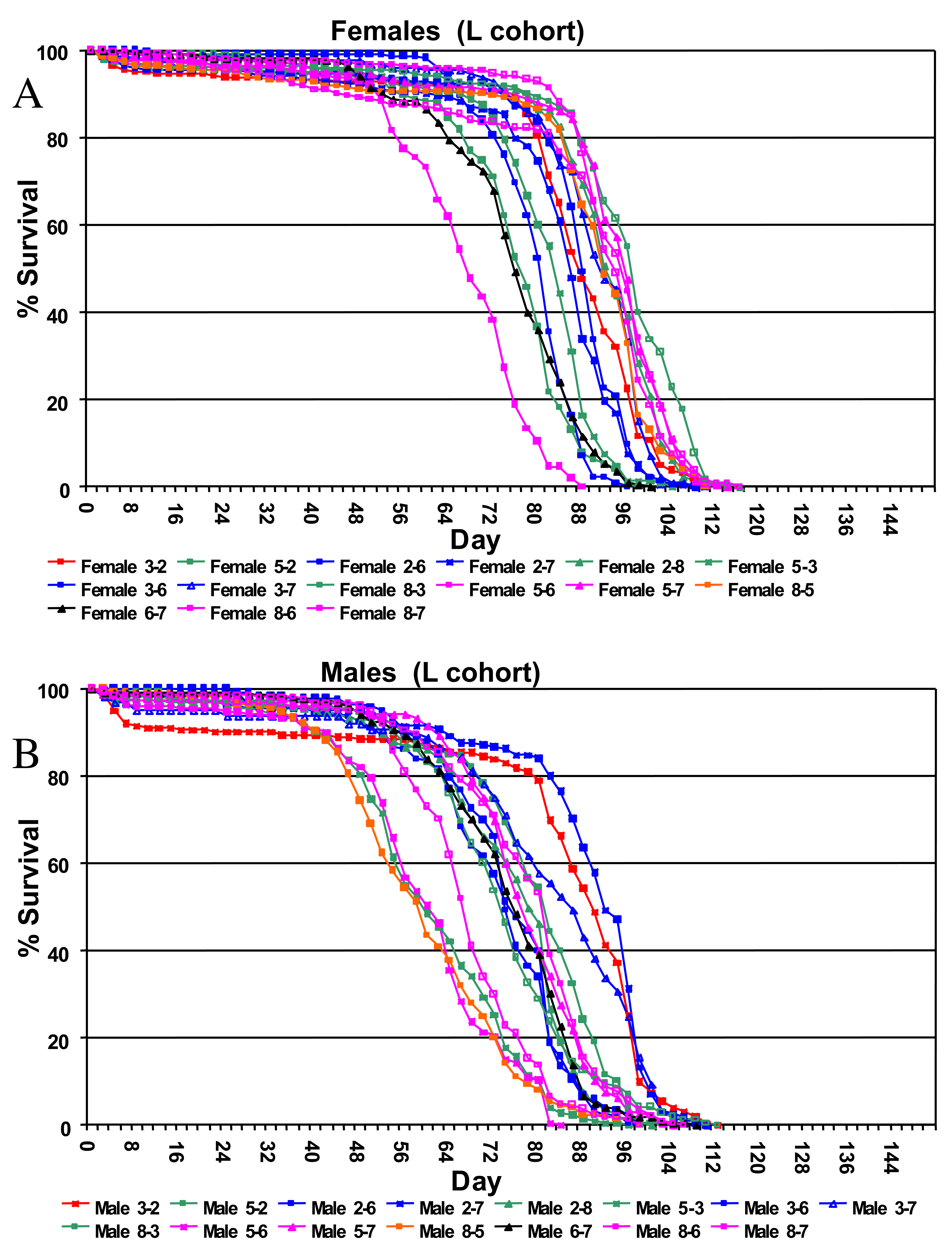

Supplement: Supplementary Figure 2C — Survival curves. (C) Box plot presentation of survival data for each genotype in cohort L. Blue boxes indicate males, pink boxes indicate females. [file aging-01-903-s002C.tif]

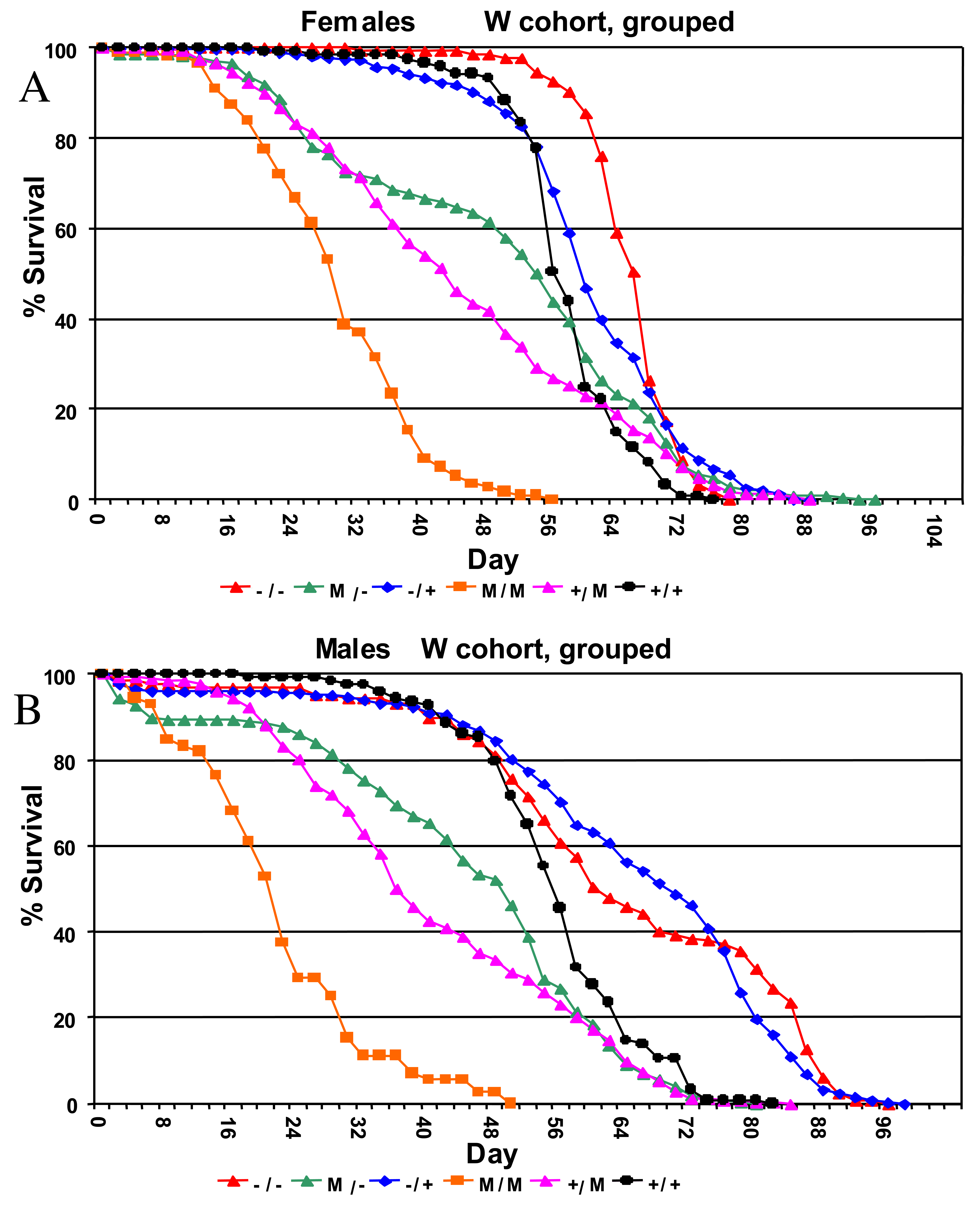

Supplement: Supplementary Figure 3 — Grouped data. (A) Females. (B) Males. [file aging-01-903-s003.tif]

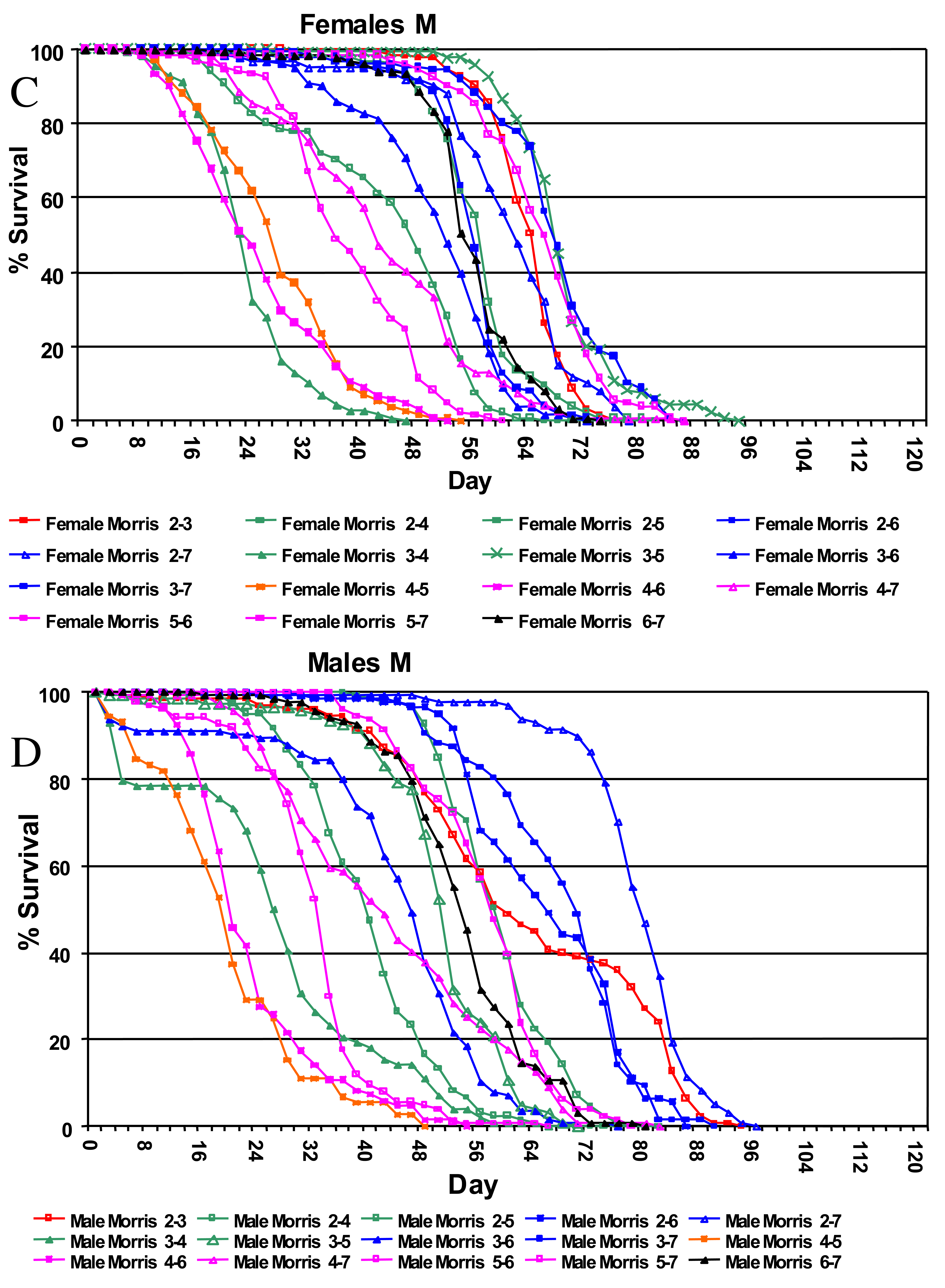

Supplement: Supplementary Figure 3C — Survival curves for each genotype. (C) Females. (D) Males [file aging-01-903-s003C.tif]

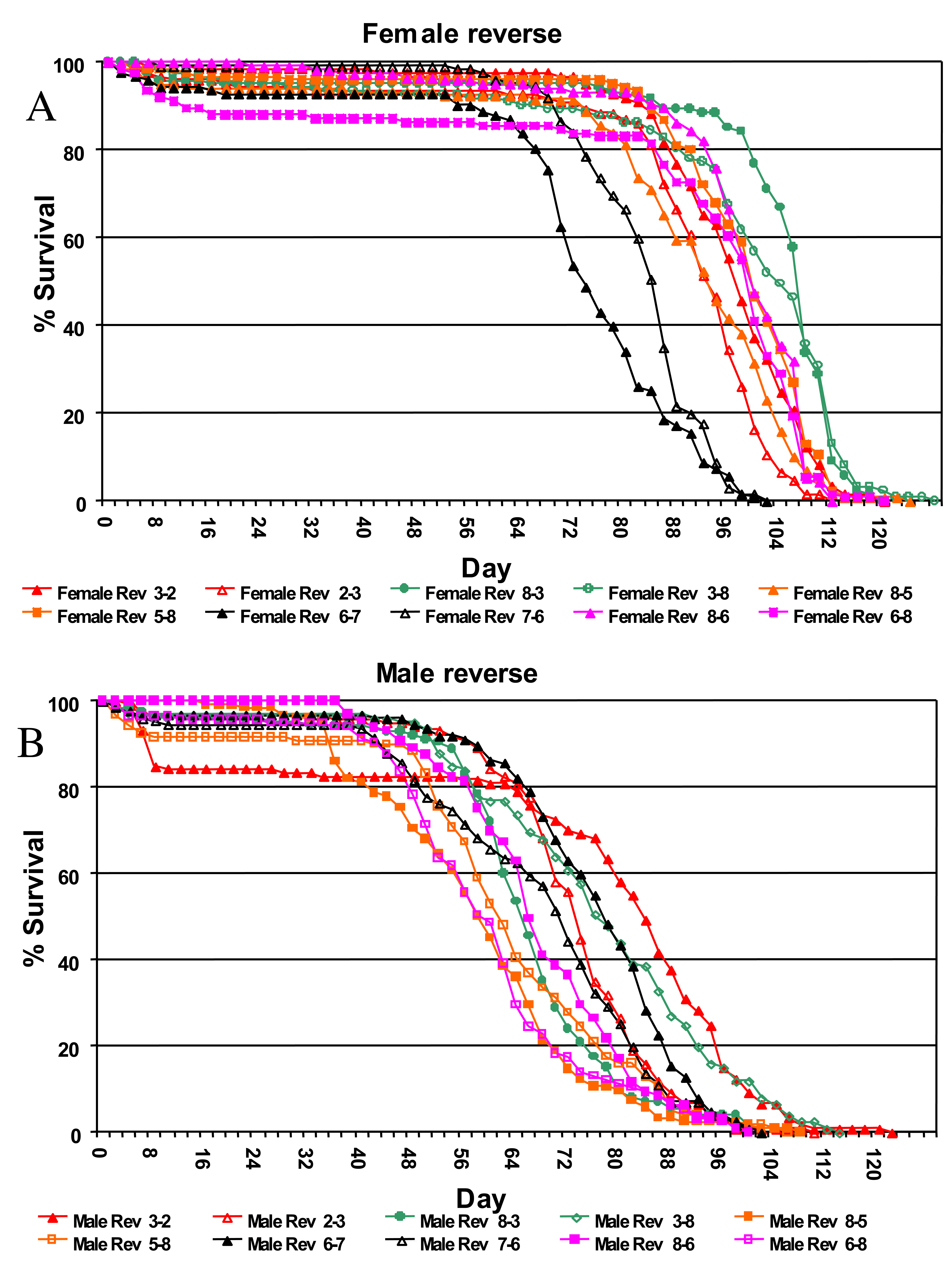

Supplement: Supplementary Figure 4 — (A) Females. (B) Males. [file aging-01-903-s004.tif]

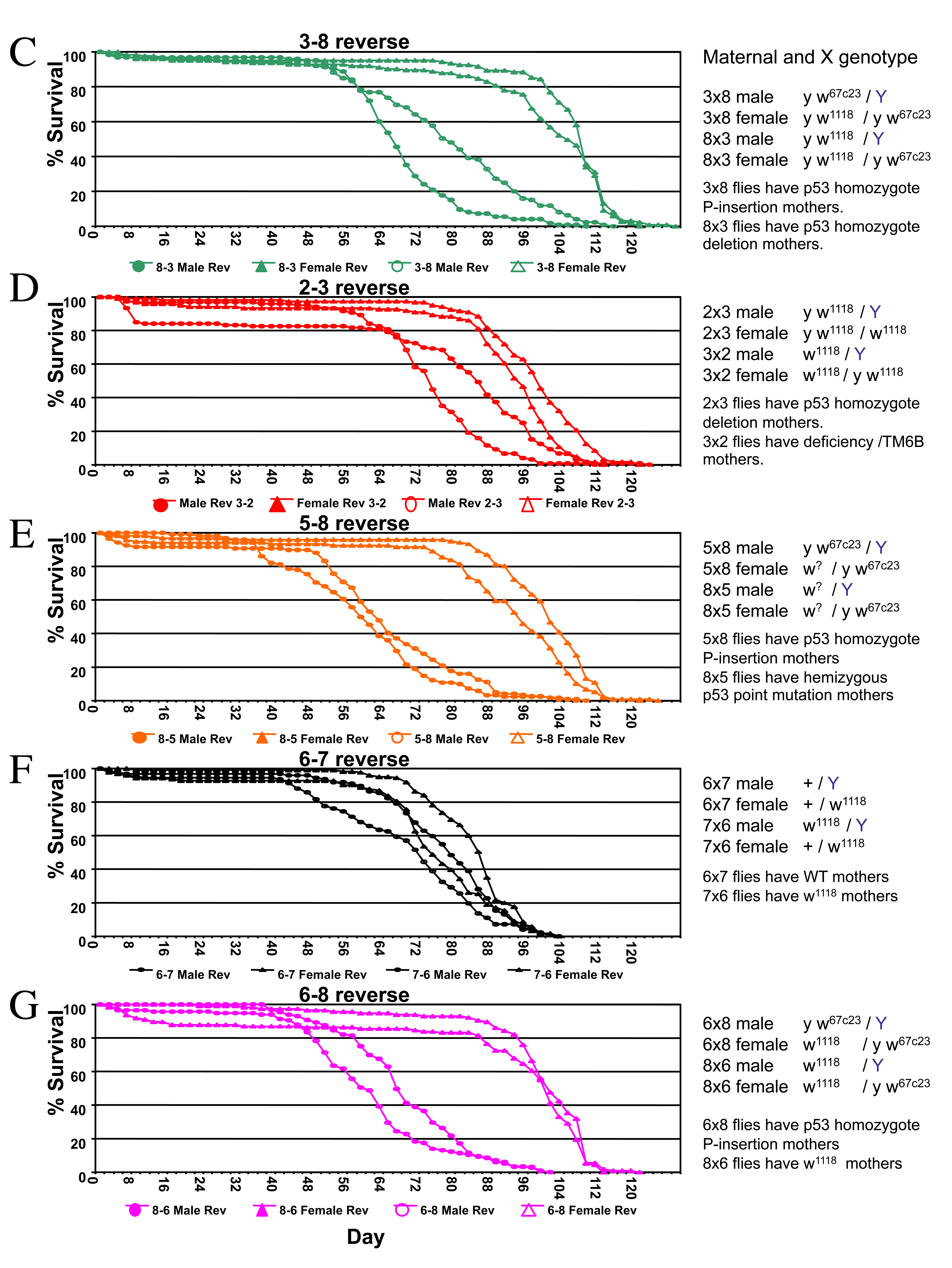

Supplement: Supplementary Figure 4C — (C-G) Comparisons of reciprocal crosses for specific genotypes. X and Y chromosomal composition of the flies is summarized to the right, along with the maternal p53 genotypes. [file aging-01-903-s004C.tif]

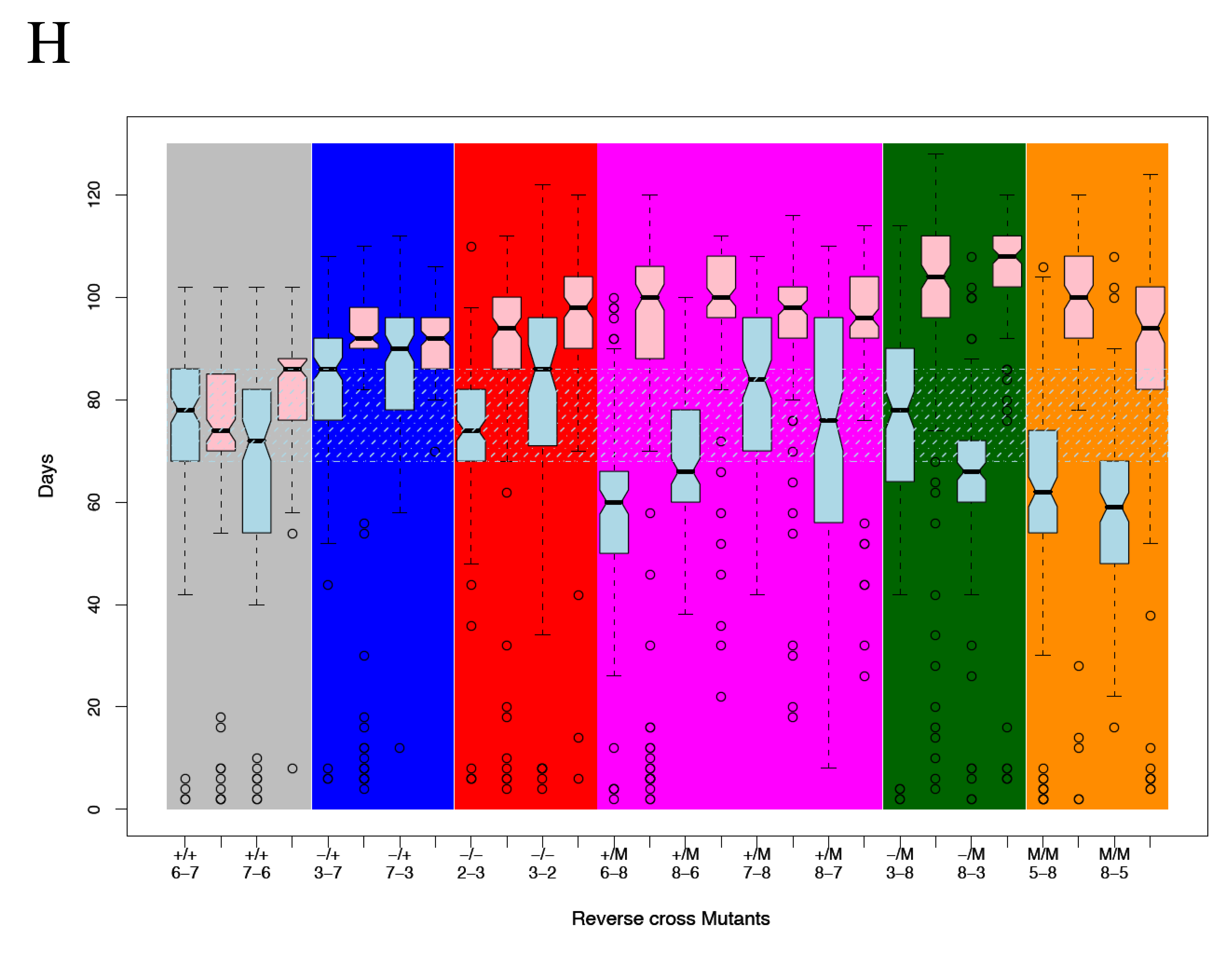

Supplement: Supplementary Figure 4H — (H) Box plot presentation of survival data for reciprocal crosses. Blue boxes indicate males, pink boxes indicate females. [file aging-01-903-s004H.tif]

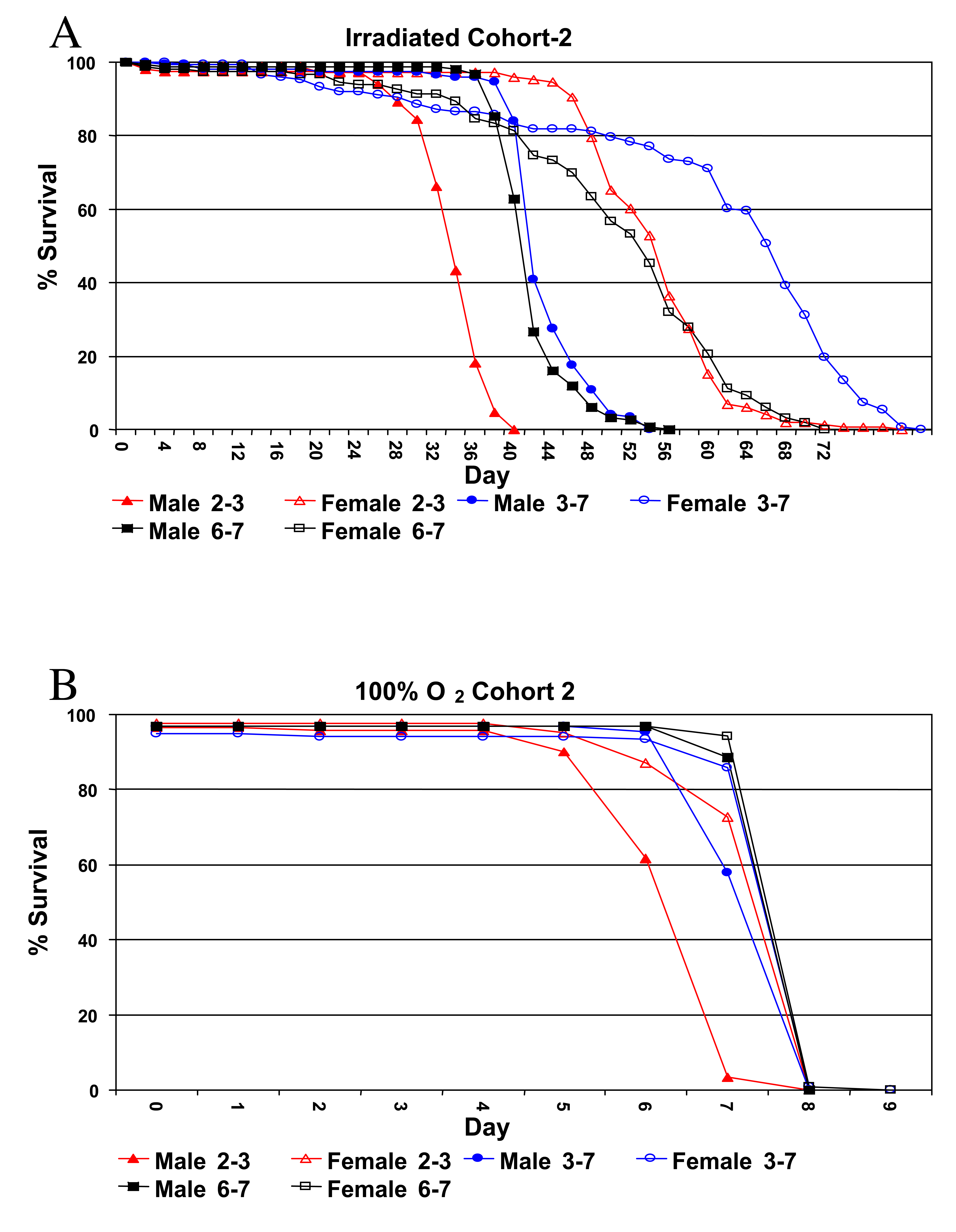

Supplement: Supplementary Figure 5 — (A) Irradiation, cohort 2. (B) 100% oxygen atmosphere, cohort 2. [file aging-01-903-s005.tif]

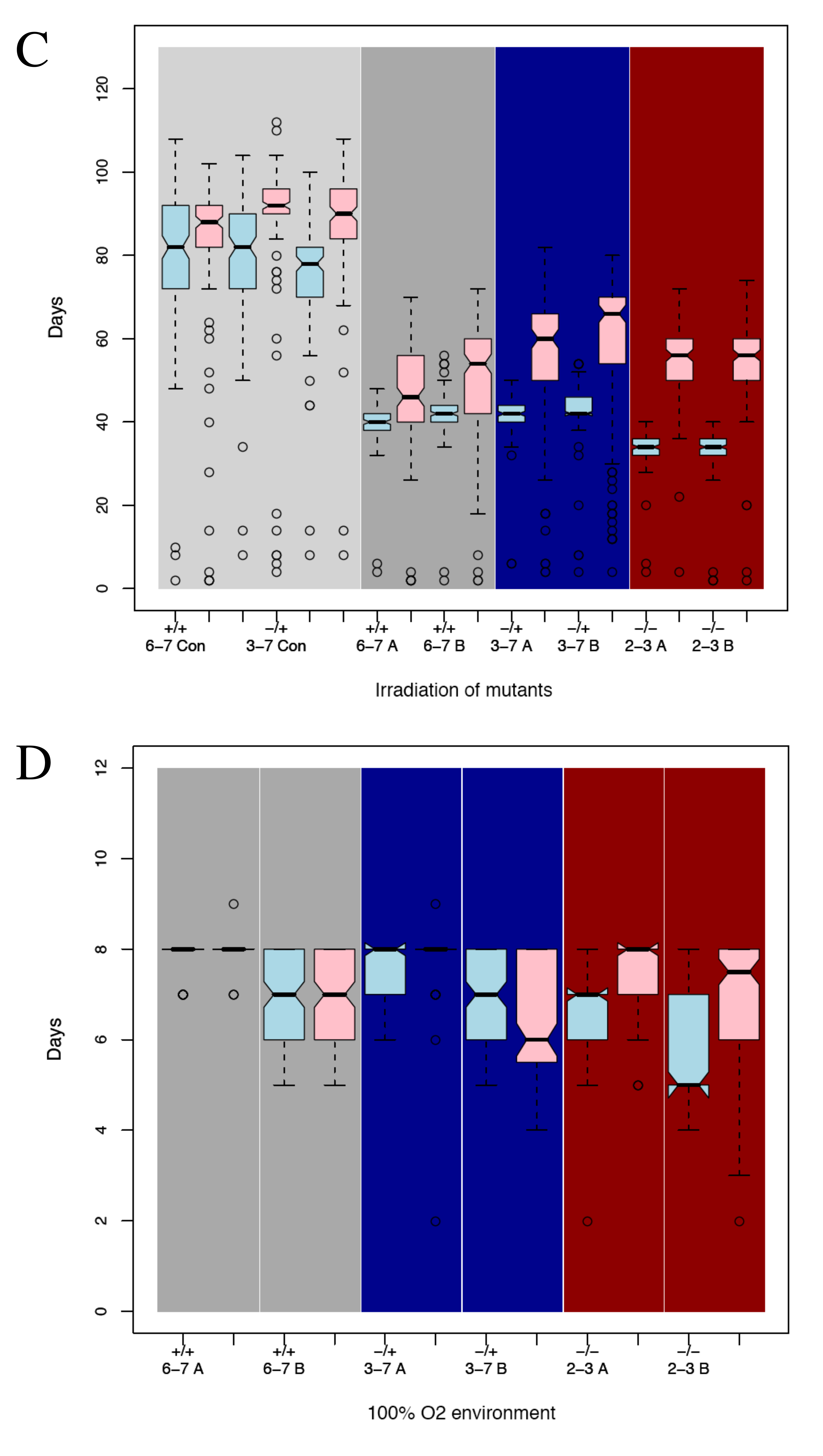

Supplement: Supplementary Figure 5C — Box plot presentation of survival data for flies subjected to stress; data is the sum of cohorts 1 and 2. (C) Irradiation. (D) 100% oxygen atmosphere. Blue boxes indicate males, pink boxes indicate females. [file aging-01-903-s005C.tif]
