# Supplementary material for: Drosophila melanogaster p53 has developmental stage-specific and sex-specific effects on adult life span indicative of sexual antagonistic pleiotropy
Source: Aging (Albany NY). 2009 Oct 27;1(11):903–36. doi: 10.18632/aging.100099 (PMC2815744; doi:10.18632/aging.100099)
Supplement: Supplementary Table 1 — 95% double bootstrap-t confidence intervals for the ratio of the means (or ratio of the percentiles) of the mutant and wild-type samples were computed as listed. The mean, median, and maximal life span values are reported for each genotype as well as the P-values representing the significance of the log-rank test of the null hypothesis that there is no difference in the probability of death between functions between wild-type untreated and p53 over-expressing flies. Note that * indicates 1.00 x10^-3^ < P < 5.00 x10^-2^, ** indicates 1.00 x10^-8^ P < 1.00 x10^-3^, *** indicates P < 1.00 x10^-8^. [file aging-01-903-s001.doc]

| **p53 GeneSwitch over-expression experiments Male** | | | | | | | | | | | |  |
| --- | --- | --- | --- | --- | --- | --- | --- | --- | --- | --- | --- | --- |
| **M-F** | **Target transgene** | **Gr** | **N** | **± SD** | **Mean life span**  **Mean CI %Δ** | | **Med life span**  **Med CI %Δ** | | **Max life span**  **Max CI %Δ** | | **P-val** | **Sig** |
| 7-9 | + | L-A- | 120 | 14.25 | 84.6 | NA | 90 | NA | 98 | NA | NA | NA |
|  |  | L+A- | 123 | 22.48 | 78.44 | −4.77 - −2.78 | 86 | −9.58 - −1.58 | 98 | −3.67 - −2.28 | 0.204 | __ |
|  |  | L-A+ | 119 | 10.94 | 83.08 | −4.76 - 1.25 | 86 | −7.34 - −1.95 | 94 | −8.41 - −1.59 | 6.58 ×10^-3^ | * |
| 16-9 | p53-WT[Ex] | L-A- | 122 | 14.76 | 71.11 | NA | 72 | NA | 86 | NA | NA | NA |
|  |  | L+A- | 0^⊗^ | NA | NA | NA | NA | NA | NA | NA | NA | NA |
|  |  | L-A+ | 117 | 12.54 | 75.38 | 2.36 - 10.37 | 78 | 5.83 - 15.51 | 90 | 1.67 – 10.11 | 6.97 ×10^-3^ | * |
| 18-9 | p53-Ct[AF51] | L-A- | 123 | 13.77 | 82.05 | NA | 86 | NA | 95.6 | NA | NA | NA |
|  |  | L+A- | 121 | 14.59 | 79.72 | −6.11 - 0.77 | 80 | −14.59 - −6.97 | 96 | −4.65 - 6.52 | 0.159 | _ |
|  |  | L-A+ | 118 | 11.15 | 79.71 | −5.73 - 0.55 | 78 | −12.89 - −9.30 | 96 | −4.01 - 4.57 | 0.032 | _ |
| 19-9 | p53-Ct[B440] | L-A- | 99 | 13.05 | 76.71 | NA | 78 | NA | 90 | NA | NA | NA |
|  |  | L+A- | 125 | 18.47 | 61.7 | −23.45 - −15.28 | 66 | −18.34 - −10.18 | 80 | −19.48 - −5.97 | 8.16 ×10^-12^ | *** |
|  |  | L-A+ | 127 | 16.1 | 73.1 | −8.26 - −0.79 | 76 | −6. 88 - −1.93 | 88.8 | −7.08 - 9.28 | 0.194 | __ |
| 20-9 | p53-259H | L-A- | 118 | 13.86 | 71.54 | NA | 72 | NA | 88 | NA | NA | NA |
|  |  | L+A- | 119 | 16.92 | 67.73 | −10.46 - −0.72 | 70 | −5.33 - 0.00 | 84 | −10.19 - 2.22 | 0.069 | __ |
|  |  | L-A+ | 125 | 10.41 | 68.9 | −7.11 - −0.02 | 70 | −2.77 - 1.76 | 78 | −15.77 - −5.53 | 2.11 ×10^-3^ | * |

| **p53 GeneSwitch over-expression experiments Female** | | | | | | | | | | | |  |
| --- | --- | --- | --- | --- | --- | --- | --- | --- | --- | --- | --- | --- |
| **M-F** | **Target transgene** | **Gr** | **N** | **± SD** | **Mean life span**  **Mean CI %Δ** | | **Med life span**  **Med CI %Δ** | | **Max life span**  **Max CI %Δ** | | **P-val** | **Sig** |
| 7-9 | + | L-A- | 116 | 9.64 | 92.02 | NA | 94 | NA | 102 | NA | NA | NA |
|  |  | L+A- | 124 | 8.61 | 91.97 | −3.33 - −0.72 | 94 | −2.88 - 2.79 | 106 | −1.41 - 3.62 | 2.55 ×10^-2^ | __ |
|  |  | L-A+ | 121 | 15.74 | 94.69 | 0.866 - 2.72 | 94 | −4.76 - 0.00 | 104 | 2.33 – 7.41 | 3.10 ×10^-3^ | * |
| 16-9 | p53-WT[Ex] | L-A- | 119 | 16.09 | 88.35 | NA | 94 | NA | 100 | NA | NA | NA |
|  |  | L+A- | 3^⊗^ | 5.03 | 91.33 | NA | 92 | NA | 95.2 | NA | NA | NA |
|  |  | L-A+ | 101 | 22.07 | 74.02 | −21.11 - −11.61 | 80 | −23.06 - −8.69 | 98 | −3.39- 2.09 | 2.21 ×10^-6^ | *** |
| 18-9 | p53-Ct[AF51] | L-A- | 123 | 8.861 | 92.2 | NA | 94 | NA | 102 | NA | NA | NA |
|  |  | L+A- | 123 | 20.08 | 81.82 | −14.90 - −8.03 | 86 | −12.87 - −5.66 | 100 | −5.59 - −1.96 | 3.40 ×10^-3^ | * |
|  |  | L-A+ | 125 | 16.53 | 89.18 | −6.71 - −0.35 | 94 | 0.00 - 3.19 | 104 | −3.83 - 2.28 | 8.57 ×10^-1^ | __ |
| 19-9 | p53-Ct[B440] | L-A- | 127 | 13.54 | 86.3 | NA | 90 | NA | 98 | NA | NA | NA |
|  |  | L+A- | 125 | 22.35 | 64.56 | −29.62 - −20.83 | 70 | −26.06 - −18.68 | 85.2 | −16.43 - −7.00 | 0 | *** |
|  |  | L-A+ | 125 | 14.39 | 89.31 | 1.96 - 6.81 | 94 | 4.38 - 9.12 | 100 | 0.35 - 4.09 | 4.72 ×10^-2^ | __ |
| 20-9 | p53-259H | L-A- | 119 | 8.495 | 75.39 | NA | 76 | NA | 84 | NA | NA | NA |
|  |  | L+A- | 125 | 22.02 | 70.24 | −11.94 - −2.49 | 76 | −3.71 - 5.72 | 88 | 1.80 - 8.56 | 2.02 ×10^-1^ | __ |
|  |  | L-A+ | 119 | 10.98 | 80.66 | 4.09 - 9.72 | 82 | 4.52 - 14.04 | 92 | 4.26 - 13.18 | 4.05 ×10^-8^ | *** |
