# Supplementary material for: Drosophila melanogaster p53 has developmental stage-specific and sex-specific effects on adult life span indicative of sexual antagonistic pleiotropy
Source: Aging (Albany NY). 2009 Oct 27;1(11):903–36. doi: 10.18632/aging.100099 (PMC2815744; doi:10.18632/aging.100099)
Supplement: Supplementary Table 2 — Wild-type p53 over-expression was induced using the GeneSwitch system and titrated at various levels with the drug RU486. Note that for the 1:1 dilution, only 1 male pupae enclosed. 95% double bootstrap-t confidence intervals for the ratio of the means (or ratio of the percentiles) of the mutant and wild-type samples in each condition were computed as listed for each p53 concentration in the combined data from two trials. The mean, median, and maximal lifespan values are reported for each genotype as well as the P-values representing the significance of the log-rank test of the null hypothesis that there is no difference in the probability of death between functions between wild-type untreated and p53 over-expressing flies. Note that * indicates 1.00 x10^-3^ < P < 5.00 x10^-2^, ** indicates 1.00 x10^-8^ P < 1.00 x10^-3^, *** indicates P < 1.00 x10^-8^. [file aging-01-903-s002.doc]

| **p53 wild-type dilution experiment, cohort 1 and cohort 2 combined, Male** | | | | | | | | | | |  | |
| --- | --- | --- | --- | --- | --- | --- | --- | --- | --- | --- | --- | --- |
| **Gr** | **M-F** | **N** | **± SD** | **Mean life span**  **Mean CI %Δ** | | **Med life span**  **Med CI %Δ** | | **Max life span**  **Max CI %Δ** | | **P-val** | | **Sig** |
| No Drug | 17-9 | 200 | 17.75 | 59.08 | NA | 64 | NA | 74 | NA | NA | | NA |
| 1:1000 | 17-9 | 204 | 9.92 | 67.68 | 10.54 – 19.30 | 68 | 3.63 - 8.99 | 78 | 2.48 – 8.52 | 4.97 ×10^-7^ | | ** |
| 1:100 | 17-9 | 209 | 11.23 | 65.13 | 6.12 – 15.09 | 68 | 6.07 - 11.26 | 74 | −4.77 - 3.03 | 1.73×10^-2^ | | * |
| 1:10 | 17-9 | 203 | 65.13 | 67.89 | 7.60 – 19.77 | 70 | 6.84 – 12.35 | 78 | 1.27 – 8.37 | 2.68 ×10^-10^ | | *** |
| 1:1 | 17-9 | 1^⊗^ | NA | NA | NA | NA | NA | NA | NA | NA | | NA |

| **p53 wild-type dilution experiment, cohort 1 and cohort 2 combined, Female** | | | | | | | | | | |  |
| --- | --- | --- | --- | --- | --- | --- | --- | --- | --- | --- | --- |
| **Gr** | **M-F** | **N** | **± SD** | **Mean life span**  **Mean CI %Δ** | | **Med life span**  **Med CI %Δ** | | **Max life span**  **Max CI %Δ** | | **P-val** | **Sig** |
| No Drug | 17-9 | 230 | 18.97 | 59.56 | NA | 68 | NA | 76 | NA | NA | NA |
| 1:1000 | 17-9 | 221 | 18.39 | 68.15 | 9.29 – 19.27 | 72 | −0.78 – 5.88 | 84 | 5.51 -10.53 | 6.66 ×10^-16^ | *** |
| 1:100 | 17-9 | 217 | 12.90 | 72.26 | 16.92 – 26.49 | 74 | 5.29 - 12.74 | 84 | 6.79 - 13.81 | 0 | *** |
| 1:10 | 17-9 | 205 | 12.40 | 67.89 | 9.33 – 18.73 | 70 | −0.33 – 5.89 | 78 | −0.52 - 4.92 | 1.35 ×10^-4^ | ** |
| 1:1 | 17-9 | 16 | 16.69 | 57.62 | −21.09 – 8.51 | 62 | −26.15 - −2.34 | 74 | −11.67 - 7.36 | 0.17 | __ |
