# Supplementary material for: Drosophila melanogaster p53 has developmental stage-specific and sex-specific effects on adult life span indicative of sexual antagonistic pleiotropy
Source: Aging (Albany NY). 2009 Oct 27;1(11):903–36. doi: 10.18632/aging.100099 (PMC2815744; doi:10.18632/aging.100099)
Supplement: Supplementary Table 3 — To assess the effect of p53 mutation on mean, median, and maximal lifespan, 95% double bootstrap t confidence intervals for the ratio of the means (or ratio of the percentiles) of the mutant and wild-type samples were computed as listed for the combined data for the L-cohort and stress assays. The log-rank test was employed to test the null hypothesis that there is no difference in the probability of death between wild-type and p53 mutant flies. P-values indicating the significance of the tests are reported. ⊗Indicates exclusion of an outlier vial. [file aging-01-903-s003.doc]

| **L cohort ^b^ Male** | | | | | | | | | | | |
| --- | --- | --- | --- | --- | --- | --- | --- | --- | --- | --- | --- |
| **M-F** | **Gr** | **N** | **± SD** | **Mean life span**  **Mean CI %Δ** | | **Med life span**  **Med CI %Δ** | | **Max life span**  **Max CI %Δ** | | **P-val** | **Sig** |
| 6-7 | +/+ | 234 | 14.82 | 74.05 | NA | 76 | NA | 88 | NA | NA | NA |
| 3-2 | -/- | ^⊗^178 | 24.53 | 83.07 | 7.13 - 16.53 | 90 | 14.26 - 23.92 | 98 | 8.84 - 11.36 | 0 | *** |
| 2-6 | -/+ | ^⊗^210 | 13.03 | 73.97 | −3.15 - 2.80 | 77 | −6.11 - 4.74 | 86.2 | −5.04 - −2.27 | 0.39 | ___ |
| 2-7 | -/+ | 195 | 19.83 | 86.86 | 13.22 - 21.24 | 92 | 14.31 - 24.91 | 100 | 11.88 - 15.32 | 0 | *** |
| 3-6 | -/+ | 236 | 13.19 | 73.69 | −3.26 - 2.47 | 74 | −6.92 - 0.51 | 88 | −1.80 - 2.91 | 0.13 | ___ |
| 3-7 | -/+ | 97 | 23.28 | 80.29 | 2.31 - 13.61 | 86 | 6.61 - 19.99 | 100 | 10.76 - 15.32 | 6.47 ×10^-10^ | *** |
| 5-6 | M/+ | 211 | 16.95 | 58.93 | −23.65 - −17.35 | 62 | −22.11 - −11.32 | 82 | NA | 0 | *** |
| 5-7 | M/+ | 187 | 14.93 | 76.47 | −0.25 - 6.59 | 78 | −0.35 - 7.45 | 90.8 | 2.35 - 8.69 | 0.074 | ___ |
| 8-6 | M/+ | 241 | 13.77 | 66.85 | −12.58 - −6.90 | 68 | −13.15 - −5.58 | 82 | −7.74 - −5.90 | 1.32 ×10^-11^ | *** |
| 8-7 | M/+ | 231 | 17.79 | 76.44 | −0.36 - 6.88 | 82 | 4.69 - 12.06 | 92 | 0.46 - 7.46 | 3.20 ×10^-3^ | * |
| 2-8 | -/M | 227 | 17.53 | 73.43 | −4.19 - 2.36 | 78 | −3.37 - 5.65 | 88 | −1.78 - 2.03 | 0.92 | ____ |
| 5-3 | M/- | 202 | 16.83 | 60.09 | −22.03 - −15.76 | 60 | −26.13 - −14.92 | 81.8 | −6.82 - −1.26 | 0 | *** |
| 5-2 | M/- | 235 | 16.79 | 78.09 | −5.00 - 1.51 | 82 | 4.83 - 11.49 | 95.2 | 9.10 - 15.26 | 6.94 ×10^-6^ | ** |
| 8-3 | M/- | 211 | 17.47 | 72.72 | −4.98 - 2.52 | 74 | −5.63 - 0.65 | 92 | −2.43 - 11.30 | 0.92 | ___ |
| 8-5 | M/M | 226 | 16.05 | 59.09 | −23.01 - −17.26 | 60 | −23.30 - −15.94 | 78 | −16.17 - −7.75 | 0 | *** |

| **L cohort ^b^ Female** | | | | | | | | | | | |
| --- | --- | --- | --- | --- | --- | --- | --- | --- | --- | --- | --- |
| **M-F** | **Gr** | **N** | **± SD** | **Mean life span**  **Mean CI %Δ** | | **Med life span**  **Med CI %Δ** | | **Max life span**  **Max CI %Δ** | | **P-val** | **Sig** |
| 6-7 | +/+ | 238 | 14.54 | 74.68 | NA | 76 | NA | 90 | NA | NA | NA |
| 3-2 | -/- | 242 | 22.22 | 84.47 | 9.00 - 16.99 | 88 | 13.07 - 21.08 | 102 | 10.47 - 19.52 | 0 | *** |
| 2-6 | -/+ | 237 | 9.92 | 79.11 | 3.40 - 8.62 | 82 | 7.89 - 13.22 | 88 | −4.63 - −0.15 | 0.05 | * |
| 2-7 | -/+ | 238 | 20.11 | 81.39 | 5.29 - 12.64 | 86 | 10.36 - 16.84 | 96 | 3.85 - 8.92 | 0 | *** |
| 3-6 | -/+ | 225 | 19.67 | 84.29 | 8.96 - 16.55 | 88 | 12.47 - 19.58 | 96 | 2.24 - 8.88 | 0 | *** |
| 3-7 | -/+ | 126 | 14.82 | 89.03 | 15.20 - 22.93 | 92 | 15.58 - 28.32 | 100 | 8.41 - 13.49 | 0 | *** |
| 5-6 | M/+ | 212 | 15.32 | 65.82 | −14.91 - −8.90 | 68 | −13.10 - −7.01 | 81.8 | −10.96 - −4.47 | 6.53 ×10^-14^ | *** |
| 5-7 | M/+ | 227 | 19.18 | 91.32 | 18.39 - 26.10 | 96 | 23.53 - 30.09 | 106 | 15.00 - 21.18 | 0 | *** |
| 8-6 | M/+ | ^⊗^208 | 21.00 | 89.36 | 15.18 - 23.80 | 96 | 23.93 - 31.21 | 106 | 14.98 - 22.09 | 0 | *** |
| 8-7 | M/+ | 210 | 15.27 | 92 | 19.74 - 26.56 | 94 | NA | 104 | NA | 0 | *** |
| 2-8 | -/M | 215 | 20.74 | 89.01 | 15.09 - 23.10 | 94 | 23.68 - 30.52 | 102 | 8.87 - 15.67 | 0 | *** |
| 5-3 | M/- | 225 | 18.15 | 78.90 | 1.89 - 9.10 | 84 | 10.53 - 13.95 | 92 | −0.165 - 4.88 | 4.28 ×10^-7^ | ** |
| 5-2 | M/- | 221 | 16.31 | 74.37 | −3.69 - 2.68 | 78 | −0.026 - 8.22 | 88 | −4.65 - 0.11 | 0.95 | ___ |
| 8-3 | M/- | 231 | 17.70 | 93.8 | 21.72 - 29.48 | 98 | 28.95 - 33.40 | 102 | 16.92 - 22.64 | 0 | *** |
| 8-5 | M/M | 231 | 22.60 | 86.94 | 12.14 - 20.48 | 92 | 18.42 - 25.00 | 102 | 10.14 - 15.88 | 0 | *** |
