# Supplementary material for: Drosophila melanogaster p53 has developmental stage-specific and sex-specific effects on adult life span indicative of sexual antagonistic pleiotropy
Source: Aging (Albany NY). 2009 Oct 27;1(11):903–36. doi: 10.18632/aging.100099 (PMC2815744; doi:10.18632/aging.100099)
Supplement: Supplementary Table 4 — To assess the effect of p53 mutation on mean, median, and maximal lifespan, 95% double bootstrap t confidence intervals for the ratio of the means (or ratio of the percentiles) of the mutant and wild-type samples were computed as listed for the grouped L-cohort data. The mean, median, and maximal lifespan values are reported for each genotype as well as the P-values for the log-rank test of the null hypothesis of identical survival functions between wild-type and p53 mutant flies. Note that * indicates 1.00 x10^-3^ < P < 5.00 x10^-2^, ** indicates 1.00 x10^-8^ P < 1.00 x10^-3^, *** indicates P < 1.00 x10^-8^. ⊗Indicates exclusion of an outlier vial. [file aging-01-903-s004.doc]

| **L cohort grouped Male** | | | | | | | | | | |
| --- | --- | --- | --- | --- | --- | --- | --- | --- | --- | --- |
| **Gr** | **N** | **± SD** | **Mean lifespan**  **Mean CI %Δ** | | **Med life span**  **Med CI %Δ** | | **Max life span**  **Max CI %Δ** | | **P-val** | **Sig** |
| +/+ | 234 | 14.82 | 74.05 | NA | 76 | NA | 88 | NA | NA | NA |
| -/- | ^⊗^178 | 24.53 | 83.07 | 4.92 - 14.50 | 90 | 13.09 - 22.05 | 98 | 8.84 - 11.36 | 0 | *** |
| -/+ | ^⊗^738 | 17.60 | 78.12 | 2.15 - 7.53 | 82 | 4.53 - 12.49 | 98 | 11.36 - 12.39 | 2.04 ×10^-8^ | *** |
| +/M | 870 | 17.48 | 69.54 | −8.41 - −3.61 | 72 | −7.82 -0.18 | 88 | −1.20 - 0.00 | 5.00 ×10^-3^ | * |
| -/M | 875 | 18.35 | 71.43 | −5.86 - −0.91 | 74 | −7.57 -0.29 | 90 | −0.28 - 3.94 | 0.73 | __ |
| M/M | 226 | 16.05 | 49.09 | −23.06 - −17.11 | 60 | −23.27 - −15.95 | 78 | −16.07 - −8.02 | 0 | *** |
