# Supplementary material for: Drosophila melanogaster p53 has developmental stage-specific and sex-specific effects on adult life span indicative of sexual antagonistic pleiotropy
Source: Aging (Albany NY). 2009 Oct 27;1(11):903–36. doi: 10.18632/aging.100099 (PMC2815744; doi:10.18632/aging.100099)
Supplement: Supplementary Table 5 — ANOVA results for differences in mean life span in Drosophila with differing p53 mutation types, where the main effect is the mutation type, comprised of grouped genotypes, and replicate vials are treated as a random effect in males (a) and females (c). Similar tests were also performed where the main effect is genotype in males (b) and females (d). Significant differences in group means were identified using Tukey's Honestly Significant Difference (HSD) multiple comparison and adjusted p-values based on the single-step method are reported for the relevant comparisons of various mutation types to wild-type. [file aging-01-903-s005.doc]

| **a. Mutation type Male** | | | | | | | |
| --- | --- | --- | --- | --- | --- | --- | --- |
| **Effects** | **DF Num** | **DF**  **Den** | **F-val** | **P-val** | **Sig** |  | |
| (Intercept) | 1 | 3106 | 23222.526 | <0.0001 | *** |  | |
| Mutation type | 5 | 3106 | 57.277 | <0.0001 | *** |  | |
| **Mutation type** | **Coef** | **DF** | **Std.Error** | **t-value** | **P-val** | **Adj P-val** | **Sig** |
| (+/+) (Intercept) | 74.033 | 3106 | 1.223 | 60.543 | <<0.0001 |  | *** |
| (-/+) | 4.065 | 3106 | 1.345 | 3.023 | 0.0025 | 0.028 | * |
| (-/-) | 8.831 | 3106 | 1.787 | 4.943 | <<0.0001 | <0.001 | *** |
| (M/+) | -4.521 | 3106 | 1.320 | -3.426 | 0.0006 | 0.007 | ** |
| (M/-) | -2.610 | 3106 | 1.320 | -1.978 | 0.0480 | 0.338 | -- |
| (M/M) | -14.940 | 3106 | 1.672 | -8.938 | <<0.0001 | <0.001 | *** |

| **b. Genotype Male** | | | | | | | |
| --- | --- | --- | --- | --- | --- | --- | --- |
| **Effects** | **DF num** | **DF den** | **F-val** | **P-val** | **Sig** |  |  |
| (Intercept) | 1 | 3097 | 27262.577 | <0.0001 | *** |  |  |
| Mutation type | 14 | 3097 | 49.205 | <0.0001 | *** |  |  |
| **Genotype** | **Coef** | **DF** | **Std.Error** | **t-value** | **P-val** | **Adj P-val** | **Sig** |
| 6-7 (intercept) | 74.0435 | 3097 | 1.153 | 64.194 | <<0.0001 |  | *** |
| 3-2 | -0.139 | 3097 | 1.614 | -0.086 | 0.931 | 1.000 | -- |
| 2-6 | -0.362 | 3097 | 1.565 | -0.231 | 0.817 | 1.000 | -- |
| 2-7 | 12.803 | 3097 | 1.647 | 7.775 | <<.0001 | <0.001 | *** |
| 3-6 | 6.501 | 3097 | 2.061 | 3.154 | 0.002 | 0.0186 | * |
| 3-7 | 8,860 | 3097 | 1.691 | 5.238 | <<0.0001 | <0.001 | *** |
| 5-6 | -15.088 | 3097 | 1.611 | -9.364 | <<0.0001 | <0.001 | *** |
| 5-7 | -7.187 | 3097 | 1.557 | -4.615 | <<0.0001 | <0.001 | *** |
| 8-6 | 2.288 | 3097 | 1.666 | 1.373 | 0.170 | 0.840 | -- |
| 8-7 | 2.397 | 3097 | 1.573 | 1.523 | 0.128 | 0.694 | -- |
| 2-8 | 4.064 | 3097 | 1.568 | 2.593 | 0.010 | 0.0931 | -- |
| 5-3 | -13.976 | 3097 | 1.632 | -8.566 | <<0.0001 | <0.001 | *** |
| 5-2 | -1.370 | 3097 | 1.612 | -0.850 | 0.395 | 0.993 | -- |
| 8-3 | -0.646 | 3097 | 1.581 | -0.409 | 0.683 | 1.000 | -- |
| 8-5 | -14.952 | 3097 | 1.583 | -9.448 | <<0.0001 | <0.001 | *** |

| **c. Mutation type Female** | | | | | | | |
| --- | --- | --- | --- | --- | --- | --- | --- |
| **Effects** | **DF Num** | **DF**  **Den** | **F-val** | **P-val** | **Sig** |  | |
| (Intercept) | 1 | 3271 | 37307.36 | <0.0001 | *** |  | |
| Mutation type | 5 | 3271 | 12.41 | <0.0001 | *** |  | |
| **Mutation type** | **Coef** | **DF** | **Std.Error** | **t-value** | **P-val** | **Adj P-val** | **Sig** |
| (+/+) (Intercept) | 74.667 | 3271 | 1.294 | 57.726 | <<0.0001 | <0.001 | *** |
| (-/+) | 8.040 | 3271 | 1.437 | 5.595 | <<0.0001 | <0.001 | *** |
| (-/-) | 9.817 | 3271 | 1.783 | 5.506 | <<0.0001 | <0.001 | *** |
| (M/+) | 10.068 | 3271 | 1.431 | 7.034 | <<0.0001 | <0.001 | *** |
| (M/-) | 9.412 | 3271 | 1.425 | 6.605 | <<0.0001 | <0.001 | *** |
| (M/M) | 12.282 | 3271 | 1.804 | 6.810 | <<0.0001 | <0.001 | *** |

| **d. Genotype Female** | | | | | | | |
| --- | --- | --- | --- | --- | --- | --- | --- |
| **Effects** | **DF num** | **DF den** | **F-val** | **P-val** | **Sig** |  |  |
| (Intercept) | 1 | 3262 | 28493.444 | <0.0001 | *** |  |  |
| Mutation type | 14 | 3262 | 40.714 | <0.0001 | *** |  |  |
| **Genotype** | **Coef** | **DF** | **Std.Error** | **t-value** | **P-val** | **Adj P-val** | **Sig** |
| 6-7 (intercept) | 74.662 | 3262 | 1.240 | 60.228 | <<0.0001 |  | *** |
| 3-2 | 9.601 | 3262 | 1.694 | 5.669 | <<0.0001 | <0.001 | *** |
| 2-6 | 4.442 | 3262 | 1.671 | 2.658 | 0.008 | 0.0776 | -- |
| 2-7 | 6.714 | 3262 | 1.669 | 4.038 | 0.0001 | <0.001 | *** |
| 3-6 | 14.751 | 3262 | 2.017 | 7.312 | <<0.0001 | <0.001 | *** |
| 3-7 | 9.823 | 3262 | 1.662 | 5.909 | <<0.0001 | <0.001 | *** |
| 5-6 | -8.906 | 3262 | 1.721 | -5.172 | <<0.0001 | <0.001 | *** |
| 5-7 | 14.672 | 3262 | 1.731 | 8.478 | <<0.0001 | <0.001 | *** |
| 8-6 | 16.652 | 3262 | 1.689 | 9.856 | <<0.0001 | <0.001 | *** |
| 8-7 | 17.510 | 3262 | 1.726 | 10.146 | <<0.0001 | <0.001 | *** |
| 2-8 | -0.291 | 3262 | 1.701 | -0.171 | 0.8644 | 1.000 | -- |
| 5-3 | 4.183 | 3262 | 1.694 | 2.470 | 0.0136 | 0.124 | -- |
| 5-2 | 19.126 | 3262 | 1.682 | 11.372 | <<0.0001 | <0.001 | *** |
| 8-3 | 14.410 | 3262 | 1.714 | 8.410 | <<0.0001 | <0.001 | *** |
| 8-5 | 12.294 | 3262 | 1.682 | 7.309 | <<0.0001 | <0.001 | *** |
