# Supplementary material for: Drosophila melanogaster p53 has developmental stage-specific and sex-specific effects on adult life span indicative of sexual antagonistic pleiotropy
Source: Aging (Albany NY). 2009 Oct 27;1(11):903–36. doi: 10.18632/aging.100099 (PMC2815744; doi:10.18632/aging.100099)
Supplement: Supplementary Table 6 — To assess the effect of p53 mutation on mean, median, and maximal lifespan, 95% double bootstrap t confidence intervals for the ratio of the means (or ratio of the percentiles) of the mutant and wild-type samples were computed as listed for the W-cohort. The log-rank test was employed to test the null hypothesis that there is no difference in the probability of death between wild-type and p53 mutant flies. P-values indicating the significance of the tests are reported. [file aging-01-903-s006.doc]

| **W cohort ^c^ Male** | | | | | | | | | | | |
| --- | --- | --- | --- | --- | --- | --- | --- | --- | --- | --- | --- |
| **M-F** | **Gr** | **N** | **± SD** | **Mean life span**  **Mean CI %Δ** | | **Med life span**  **Med CI %Δ** | | **Max life span**  **Max CI %Δ** | | **P-val** | **Sig** |
| 6-7 | +/+ | 123 | 10.48 | 53.64 | NA | 54 | NA | 69.2 | NA | NA | NA |
| 2-3 | -/- | 125 | 18.89 | 63.2 | 11.33 - 24.50 | 60 | 1.89 – 21.56 | 86 | 6.72 – 26.42 | 2.62 ×10^-10^ | *** |
| 2-6 | -/+ | 122 | 11.7 | 66.18 | 18.72 - 28.60 | 66 | 12.94 – 30.20 | 80 | 1.64 -18.95 | 0 | *** |
| 2-7 | -/+ | 130 | 9.27 | 79.11 | 42.19 -52.75 | 80 | 41.55 – 54.53 | 88 | 9.94 -29.94 | 0 | *** |
| 3-6 | -/+ | 114 | 15.55 | 43.33 | −24.71 - −14.15 | 46 | −20.74 - −11.38 | 57.4 | −27.18 - −12.22 | 1.30 ×10^-8^ | ** |
| 3-7 | -/+ | 127 | 11.33 | 67.54 | 21.00 - 30.88 | 70 | 23.40 – 34.64 | 78.8 | 0.34 – 19.58 | 0 | *** |
| 4-6 | M/+ | 120 | 9.617 | 23.3 | −59.34 - −53.76 | 20 | −65.84 - −61.16 | 38 | −53.86 - −35.26 | 0 | *** |
| 4-7 | M/+ | 119 | 15.74 | 43.14 | −24.28 - −13.92 | 42 | −29.24 - −14.46 | 66 | −17.60 - −2.42 | 1.60 ×10^-4^ | ** |
| 5-6 | M/+ | 124 | 9.25 | 32.02 | −43.68 - −37.22 | 34 | −39.34 - −31.90 | 40 | −51.62 - −38.42 | 0 | *** |
| 5-7 | M/+ | 126 | 9.38 | 56.98 | 2.01 - 10.35 | 58 | 2.87 – 11.76 | 68 | −14.53 - 1.27 | 0.038 | * |
| 2-4 | -/M | 120 | 9.66 | 39.97 | −28.86 - −22.20 | 40 | −30.94 - −22.77 | 52 | −35.37 - −22.50 | 0 | *** |
| 2-5 | -/M | 125 | 7.96 | 59.12 | 6.31 – 14.34 | 60 | 6.94 – 19.48 | 70 | −12.64 - 2.11 | 7.69 ×10^-4^ | ** |
| 3-4 | -/M | 98 | 15.58 | 26.53 | −55.51 - −45.30 | 27 | −57.20 - −49.80 | 50 | −37.31 - −22.61 | 0 | *** |
| 3-5 | -/M | 120 | 10.56 | 50.28 | −10.41 - −2.46 | 52 | −7.44 - 3.31 | 62 | −20.46 - −6.79 | 1.32 ×10^-3^ | * |
| 4-5 | M/M | 72 | 10.77 | 19.89 | −66.79 - −58.37 | 20 | −64.24 - −58.24 | 35.4 | −57.32 - −28.56 | 0 | *** |
