# Supplementary material for: Drosophila melanogaster p53 has developmental stage-specific and sex-specific effects on adult life span indicative of sexual antagonistic pleiotropy
Source: Aging (Albany NY). 2009 Oct 27;1(11):903–36. doi: 10.18632/aging.100099 (PMC2815744; doi:10.18632/aging.100099)
Supplement: Supplementary Table 7 — a Mean life span, days +/- SD. b Median life span, days +/- SD Life Span, days. [file aging-01-903-s007.doc]

| Group | Genotype | Sex | N | N Median Life span phenotype^b^ | Log Rank (vs +/+) p |  |
| --- | --- | --- | --- | --- | --- | --- |
| W cohort |  |  |  |  |  |  |
|  | -/- | Male | 127 | 63.2±20.25 | 60±20.66 | 56±8.10 |
|  | M/- | Male | 492 | 44.68±18.88 | 48±19.23 | 5.65e-05 |
|  | +/- | Male | 504 | 64.65±19.73 | 68±19.91 | 0.00 |
|  | M/M | Male | 72 | 19.89±10.84 | 20±10.82 | 0.00 |
|  | +/M | Male | 492 | 39.02±17.21 | 36±17.78 | 8e-09 |
|  | +/+ | Male | 123 | 53.64±10.58 | 54±10.52 |  |
|  |  |  |  |  |  |  |
|  | -/- | Female | 125 | 63.9±6.49 | 66±6.18 | 2.22e-16 |
|  | M/- | Female | 490 | 48.27±19.90 | 54±20.34 | 0.947 |
|  | +/- | Female | 498 | 58.67±12.00 | 58±12.13 | 8.27e-07 |
|  | M/M | Female | 111 | 27.45±10.38 | 28±9.96 | 0.00 |
|  | +/M | Female | 480 | 42.82±18.56 | 42±18.67 | 0.000628 |
|  | +/+ | Female | 121 | 55.80±8.15 | 56±8.10 |  |
