# Supplementary material for: Drosophila melanogaster p53 has developmental stage-specific and sex-specific effects on adult life span indicative of sexual antagonistic pleiotropy
Source: Aging (Albany NY). 2009 Oct 27;1(11):903–36. doi: 10.18632/aging.100099 (PMC2815744; doi:10.18632/aging.100099)
Supplement: Supplementary Table 8 — The mean, median, and maximal lifespan values are reported for each genotype as well as P-values for the log-rank test of the null hypothesis of identical survival functions between wild-type (+/+; 6-7) or the reverse cross wild-type (+/+; 7-6) and p53 mutant flies are denoted by superscript a and b, respectively. ⊗ Indicates exclusion of an outlier vial. [file aging-01-903-s008.doc]

| **Reverse cross Male** | | | | | | | | | | |
| --- | --- | --- | --- | --- | --- | --- | --- | --- | --- | --- |
| **M-F** | **Gr** | **N** | **± SD** | **Mean** | **Med** | **Max** | **P-val^a^** | **Sig^a^** | **P-val^b^** | **Sig^b^** |
| 6-7 | +/+ | 124 | 17.89 | 75.19 | 78 | 92 | NA | NA | 1.96 ×10^-3^ | * |
| 7-6 | +/+ | 126 | 21.08 | 66.4 | 72 | 88 | 1.96 ×10^-3^ | * | NA | NA |
| 2-3 | -/- | 120 | 16.81 | 72.08 | 74 | 88 | 2.74 ×10^-2^ | * | 2.64 ×10^-1^ | -- |
| 3-2 | -/- | ^⊗^ 71 | 26.35 | 78.65 | 86 | 102 | 6.43 ×10^-5^ | ** | 1.58 ×10^-8^ | *** |
| 3-7 | -/+ | 131 | 16.95 | 82.15 | 86 | 98 | 7.14 ×10^-6^ | * | 2.90 ×10^-12^ | *** |
| 7-3 | -/+ | 120 | 13.94 | 85.85 | 90 | 100 | 1.11 ×10^-9^ | *** | 2.22 ×10^-16^ | *** |
| 6-8 | +/M | 115 | 18.34 | 59.04 | 60 | 83.2 | 5.35 ×10^-11^ | *** | 4.15 ×10^-4^ | ** |
| 8-6 | +/M | 129 | 13.99 | 67.64 | 66 | 84 | 2.89 ×10^-6^ | ** | 2.06 ×10^-1^ | -- |
| 7-8 | +/M | 122 | 17.02 | 81.25 | 84 | 100 | 9.69 ×10^-6^ | ** | 7.28 ×10^-11^ | *** |
| 8-7 | +/M | 117 | 22.92 | 74.97 | 76 | 102 | 3.77 ×10^-3^ | * | 3.33 ×10^-6^ | *** |
| 3-8 | -/M | 125 | 21.50 | 75.98 | 78 | 102 | 2.80 ×10^-2^ | * | 5.31 ×10^-6^ | *** |
| 8-3 | -/M | 125 | 17.39 | 65.10 | 66 | 80 | 1.92 ×10^-7^ | ** | 5.74 ×10^-2^ | -- |
| 5-8 | M/M | 119 | 22.11 | 61.28 | 62 | 88 | 1.85 ×10^-6^ | ** | 1.07 ×10^-1^ | -- |
| 8-5 | M/M | 122 | 16.77 | 58.21 | 59 | 79.6 | 1.70 ×10^-13^ | *** | 3.31 ×10^-5^ | ** |
