# Supplementary material for: Drosophila melanogaster p53 has developmental stage-specific and sex-specific effects on adult life span indicative of sexual antagonistic pleiotropy
Source: Aging (Albany NY). 2009 Oct 27;1(11):903–36. doi: 10.18632/aging.100099 (PMC2815744; doi:10.18632/aging.100099)
Supplement: Supplementary Table 9 — 95% double bootstrap-t confidence intervals for the ratio of the means (or ratio of the percentiles) of the mutant and wild-type samples in each condition were computed as listed. The mean, median, and maximal lifespan values are reported for each genotype as well as the P-values representing the significance of the log-rank test of the null hypothesis that there is no difference in the probability of death between wild-type and p53 mutant flies. [file aging-01-903-s009.doc]

| **Stress experiments Male** | | | | | | | | | | | |
| --- | --- | --- | --- | --- | --- | --- | --- | --- | --- | --- | --- |
| **M-F** | **Gr** | **N** | **± SD** | **Mean life span**  **Mean CI %Δ** | | **Med life span**  **Med CI %Δ** | | **Max life span**  **Max CI %Δ** | | **P-val** | **Sig** |
| **Standard conditions** | | | | | | | | | | | |
| 6-7 | +/+ | 129 | 17.12 | 79.81 | NA | 82 | NA | 98 | NA | NA | NA |
| 2-3 | -/- | 117 | 13.14 | 75.56 | −9.12 - −1.15 | 78 | −7.64 - −1.03 | 88 | −14.36 – 7.09 | 7.84 ×10^-6^ | ** |
| 3-7 | -/+ | 124 | 15.96 | 79.81 | −4.18 - 4.43 | 82 | −5.30 - 4.00 | 98 | −5.52 – 3.24 | 0.73 | --- |
| **Ionizing radiation** | | | | | | | | | | |  |
| 6-7 | +/+ | 274 | 5.78 | 40.82 | NA | 42 | NA | 46 | NA | NA | NA |
| 2-3 | -/- | 273 | 5.43 | 33.12 | −20.68 - −17.06 | 34 | −25.54 - −19.05 | 38 | −22.65 - – 12.41 | 0 | *** |
| 3-7 | -/+ | 273 | 6.15 | 42.39 | 1.80 - 6.00 | 42 | −7.48 - 0.00 | 47.6 | −1.24 – 11.02 | 2.02 ×10^-7^ | ** |
| **100% O2** | | | | | | | | | | |  |
| 6-7 | +/+ | 238 | 0.56 | 17.83 | NA | 18 | NA | 18 | NA | NA | NA |
| 2-3 | -/- | 232 | 1.56 | 15.16 | −16.13 - −14.10 | 16 | −11.11 - −11.11 | 16 | −11.11 - −11.11 | 0 | *** |
| 3-7 | -/+ | 244 | 1.05 | 17.16 | −4.38 - −3.05 | 18 | NaN - NaN | 18 | NaN-NaN | 4.44 ×10^-16^ | *** |
