# Supplementary material for: Drosophila melanogaster p53 has developmental stage-specific and sex-specific effects on adult life span indicative of sexual antagonistic pleiotropy
Source: Aging (Albany NY). 2009 Oct 27;1(11):903–36. doi: 10.18632/aging.100099 (PMC2815744; doi:10.18632/aging.100099)
Supplement: Supplementary Table 10 — The W cohort was cultured on "Old food" recipe, as were all flies in experiments in Tower laboratory prior to September 2005. The L cohort and all other experiments presented here were conducted using "New food" recipe. [file aging-01-903-s010.doc]

| **For One Liter** | **Old Food** | **New Food** |
| --- | --- | --- |
| Water (L) | 1 | 1 |
| Sucrose (g) | 0 | 0 |
| Dextrose (g) | 0 | 105 |
| Molasses (ml) | 100 | 0 |
| Agar (g) | 9 | 8 |
| Yeast (g) | 41 | 26 |
| Cornmeal (g) | 100 | 50 |
| Tegosept (g) | 2.5 | 1.7 |
| 95% Ethanol (ml) | 22.5 | 8.6 |
| Propionic Acid (ml) | 8 | 1.9 |
| phosphoric acid | 0 | 0 |
